# The economics of malaria control and elimination: a systematic review

**DOI:** 10.1186/s12936-016-1635-5

**Published:** 2016-12-12

**Authors:** Rima Shretta, Anton L. V. Avanceña, Arian Hatefi

**Affiliations:** 1 The Global Health Group, University of California, San Francisco, 550 16th St, 3rd Floor, Box 1224, San Francisco, CA 94158 USA; 2 Swiss Tropical and Public Health Institute, Socinstrasse 57, 4002 Basel, Switzerland; 3 University of Basel, Petersplatz 1, 4001 Basel, Switzerland; 4 Department of Medicine, School of Medicine, University of California, San Francisco, San Francisco, CA USA

## Abstract

**Background:**

Declining donor funding and competing health priorities threaten the sustainability of malaria programmes. Elucidating the cost and benefits of continued investments in malaria could encourage sustained political and financial commitments. The evidence, although available, remains disparate. This paper reviews the existing literature on the economic and financial cost and return of malaria control, elimination and eradication.

**Methods:**

A review of articles that were published on or before September 2014 on the cost and benefits of malaria control and elimination was performed. Studies were classified based on their scope and were analysed according to two major categories: cost of malaria control and elimination to a health system, and cost-benefit studies. Only studies involving more than two control or elimination interventions were included. Outcomes of interest were total programmatic cost, cost per capita, and benefit-cost ratios (BCRs). All costs were converted to 2013 US$ for standardization.

**Results:**

Of the 6425 articles identified, 54 studies were included in this review. Twenty-two were focused on elimination or eradication while 32 focused on intensive control. Forty-eight per cent of studies included in this review were published on or after 2000. Overall, the annual per capita cost of malaria control to a health system ranged from $0.11 to $39.06 (median: $2.21) while that for malaria elimination ranged from $0.18 to $27 (median: $3.00). BCRs of investing in malaria control and elimination ranged from 2.4 to over 145.

**Conclusion:**

Overall, investments needed for malaria control and elimination varied greatly amongst the various countries and contexts. In most cases, the cost of elimination was greater than the cost of control. At the same time, the benefits of investing in malaria greatly outweighed the costs. While the cost of elimination in most cases was greater than the cost of control, the benefits greatly outweighed the cost. Information from this review provides guidance to national malaria programmes on the cost and benefits of malaria elimination in the absence of data. Importantly, the review highlights the need for more robust economic analyses using standard inputs and methods to strengthen the evidence needed for sustained financing for malaria elimination.

**Electronic supplementary material:**

The online version of this article (doi:10.1186/s12936-016-1635-5) contains supplementary material, which is available to authorized users.

## Background

In the past decade and a half, remarkable progress in malaria control has been achieved with a 37% decline in malaria incidence and 60% reduction in malaria deaths globally [1]. Almost half of the world’s nations are now malaria free [2] and several countries have reduced malaria transmission to levels low enough to allow them to embark on, and in many cases achieve, elimination [3].

Despite international consensus that malaria elimination leading to global eradication is a worthwhile goal [2], sustaining domestic and international funding as the malaria burden declines is a serious concern for many countries. External aid is on the decline [4] and multilateral and bilateral donor funds are increasingly shifting away from disease-specific financing or being targeted towards low-income, high-burden countries. At the same time, domestically there is mounting competition for limited resources from other pressing disease priorities.

There is little disagreement that elimination is an attractive investment in the long term due to its ability to pay for itself through future reductions in spending and its generation of broader economic benefits. The contribution of malaria elimination to colossal health and development returns of global eradication is also implicitly recognized [5, 6]. Notwithstanding, malaria elimination requires additional front-loading of investments into robust surveillance systems to detect and respond to remaining cases. While socio-economic and other structural changes will eventually change the intrinsic baseline potential for transmission in countries such that active measures are no longer required [7], the decision facing policymakers is how to best allocate finite resources in the short term. Countries who have successfully lowered their malaria burden are faced with the risk of losing or severely reducing their recurrent expenditure for elimination and preventing the re-introduction of malaria at a critical period in the malaria elimination efforts [8]. At the same time, they face the risk of resurgence due to the persistent importation of new cases which will not only have devastating effects on the health and welfare of individuals, but will also place an additional economic burden on the health system. A review on malaria resurgence occurring from the 1930s through to the 2000s demonstrated that almost all resurgence events could be attributed, at least in part, to the weakening of malaria control programmes for a variety of reasons, of which resource constraints were the most common [9]. In addition, lessons learned from the Global Malaria Eradication Programme (GMEP), which ended in 1969, affirm that while well-funded interventions can have a major impact on the disease, such gains are fragile and can easily be reversed particularly in the short term in areas that continue to be epidemiologically and entomologically receptive and vulnerable.

The economic impact of malaria has been studied for well over a century. The numbers of such studies have escalated since the conclusion of the GMEP in the late 1960s and more so starting early 2000. Many of these studies have reported data on the economic burden of malaria and the cost of malaria programmes. However, evidence on the economics of malaria elimination remains disparate without a comprehensive synthesis of the marginal costs of elimination that can be used by policymakers for decision-making. Policymakers need to know how much it costs to achieve reductions in malaria burden and elimination, whether the cost savings of elimination will offset the initial investment given that elimination requires to avert the last few cases, and what are the financial returns of elimination versus maintaining the status quo.

Economic methods such as cost-effectiveness analysis (CEA) and cost-benefit analysis (CBA) have commonly been used to assess the comparative value of investing in malaria control interventions. CEA, which calculates the amount of funding an intervention needs to prevent loss of a standard unit of disease burden, is the most commonly used approach to compare the economic attractiveness of health programmes. In an elimination context, CEA is relevant for identifying the optimum mix of interventions needed to sustain elimination. However, it does not help drive decisions on the economic appeal of malaria elimination as a whole [10]. In addition, as the burden of malaria diminishes, elimination interventions become less cost-effective because the incremental health gains are significantly smaller compared to programme costs. Furthermore, malaria transmission becomes increasingly concentrated in small geographic areas that are often difficult, and more expensive to reach such that a a simple cost-effectiveness ratio (CER) is unlikely to be favourable [11]. When evaluated as a CER, the health and economic gains associated with elimination may already be captured by control [12]. Lastly, CERs may not fully capture all the benefits and positive externalities that malaria elimination and prevention of re-introduction (POR) may bring, particularly when considering the cost of malaria resurgence [9, 13].

To generate results most relevant to policy, malaria elimination requires a comparison of cost with a counterfactual scenario of malaria control to reflect programmatic realities. In practice, most economic analyses in malaria use a loosely defined status quo, which varies substantially but is most often that of partial control. WHO recommends a null state of disease without intervention as the counterfactual scenario. Used in several analyses, this alternative is neither pragmatic nor sustainable but can provide information to understand the benefits of continued investment in malaria when the disease is greatly reduced or absent. Others have recommended the use of controlled low-endemic malaria as the most policy-relevant alternative for economic analyses of elimination [13]. However, the threats of drug and insecticide resistance and the instability of international financing mean that malaria control may not be sustained in the long term. In addition, elimination delivers additional indirect benefits outside of health. As a country approaches and reaches elimination, other countries benefit from reduced importation of malaria conferring positive externalities to neighbouring countries as well. A comprehensive CBA enables these broader benefits to be translated into a common metric and is therefore a more effective means to inform strategic decisions.

The aim of this paper is to review the existing literature and evidence on the costs and benefits of malaria elimination. Specifically, this paper presents a comprehensive review of literature on the cost of malaria control as well as those of achieving and of sustaining elimination and the benefits generated by malaria elimination compared to the cost of malaria control. The review intends to elicit evidence along the various phases of the programme: control, elimination and POR [34].

## Methods

### Search strategy

Following PRISMA guidelines [14], a systematic search of peer-reviewed literature in English, French and Spanish, pertaining to economics of malaria, published on or before September 2014 was conducted. Databases searched were MEDLINE via PubMed, SCOPUS and Google Scholar using MeSH terms as well as other keywords. The term ‘malaria’ was combined with ‘elimination’ and ‘eradication’ and the following search terms: ‘economics’, ‘cost’, ‘cost analysis’, ‘cost allocation’, ‘cost apportionment’, ‘cost control’, ‘cost of illness’, ‘employer health costs’, ‘hospital costs’, ‘health care costs’, ‘drug costs’, ‘direct service costs’, ‘health expenditures’, ‘financing’, and ‘cost-benefit analysis’. A detailed list of search terms and corresponding results are available upon request.

Two independent database searches were carried out to ensure an exhaustive search of the literature. AA, who conducted the literature search, was blinded to the initial search strategy but used the same databases and publication timeframe. The two lists of papers were subsequently merged and duplicates were removed. Reference lists of papers that met the inclusion criteria were also screened and included 13 additional articles that were deemed relevant.

### Article screening and selection

Titles and abstracts of all initial search results were reviewed for relevance, and those that included some form of economic analysis were assessed further for eligibility. Articles that did not have abstracts available online but were thought to be relevant based on their titles alone were included in the full-text assessment. Articles were excluded during full-text assessment if they did not meet the inclusion criteria or if their full-text versions could not be located after multiple attempts. In case of a disagreement during article selection, inclusion and exclusion, data extraction, article categorization and quality appraisal, the authors discussed each case separately until a consensus was reached.

### Inclusion criteria

Articles were included if they: (a) evaluated at least three interventions, suggesting intensive control or elimination rather than individual or limited interventions; (b) presented final costs and benefits in economic or monetary terms; and, (c) provided a clear description of data sources and methodology. Micro-economic studies that assessed the cost of delivering malaria interventions to the health system were included and economic evaluations that included cost-benefit type analyses on malaria interventions were also included.

### Exclusion criteria

Studies that used preference approaches (e.g., willingness to pay) for valuing costs and benefits were excluded as a way to limit the analysis to studies that used empirical or secondary cost data rather than elicitation methods. Papers that only presented descriptive statistics or reiterated findings from other studies already included in the review were also excluded. However, any review papers that either conducted any primary analysis on scientific literature were included [10, 15].

### Data abstraction, standardization and qualitative synthesis

A standard Microsoft Excel^®^ template was used to abstract detailed information about each study’s publication year, study setting, study period, sources of data, and the outcomes of interest. Monetary data were first adjusted to US$ in the year of the initial study (if the authors had not already done so) using historical exchange rates provided in the article. If the article did not provide exchange rates, historical exchange rates were obtained from the World Bank official exchange rate database for year 1981 onwards [16] and other online sources such as OANDA [17]. For studies where the currency year was not provided, the publication date or date of article submission was used for the currency conversion. All monetary data were standardized to 2013 US$ using consumer price index conversion factors published by Oregon State University, USA [18].

Studies that assessed health system costs of malaria control and elimination were abstracted for total costs, cost per population at risk (PAR), and cost per capita. When total costs only were provided, the annual cost per capita was calculated by dividing the annual aggregate or total cost by either the PAR or total population numbers reported in the articles or their supplements published online. Similarly, the authors attempted to convert other averaged costs (e.g., cost per person protected, cost per suspected case, cost per case treated) into cost per capita whenever possible to help account for differences in intended programme coverage. It is important to note, however, that a standardized way to measure or calculate PAR does not exist [19–21] making comparisons among such reported costs potentially problematic.

For CBAs, net benefits (also referred to as net present value or net social benefit) and benefit-cost ratios (BCRs) were extracted. If net benefits or BCRs were not calculated in the original study, they were computed based on total benefits and total costs reported in the study whenever possible to facilitate comparisons among CBAs.

### Quality assessment and critical appraisal

The quality of the included studies was assessed using two checklists published in the literature. For CBAs, the ten-point Drummond checklist first developed by Drummond and colleagues in 1997 [22, 23] was adapted. Each study was assigned a total score equal to the number of ‘yes’ ratings it received out of ten questions in the checklist. For cost analysis studies, the two-point evaluation criteria developed by Fukuda and Imanaka was adapted to assess the quality and transparency of costing exercises [24]. The Fukuda and Imanaka criteria evaluated each costing study based on its clarity of scope and accuracy of costing methodology, with activity-based microcosting getting the highest score.

## Results

### Literature search

**Fig. 1:**
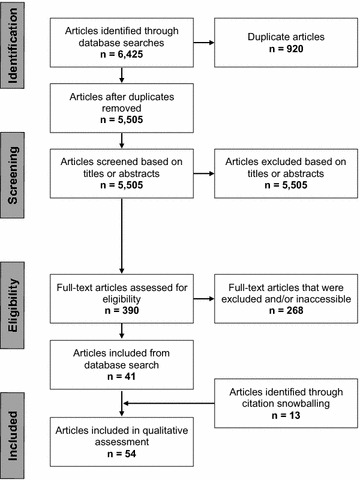
PRISMA diagram

A total of 6425 articles were identified through database searches. After removal of duplicates, 5505 titles and abstracts were initially screened, and 390 full-text articles were reviewed further for eligibility. After reviewing full-text articles, 40 from the database searches and 14 from citation snowballing were included in the final qualitative analysis (Fig. 1). Most of the studies conducted more than one type of economic analysis and therefore are not classified into mutually exclusive categories.

Of the 54 articles in this review, 22 were focused on elimination while the remaining 32 were on intensive control. Fifty-three studies estimated the programmatic costs of malaria control and elimination, and ten studies estimated both costs and benefits (Table 1).

**Table 1:** Summary of included articles

Total number of studies included in qualitative review	54	
Number of studies with more than one economic outcome reported	9	

	Total number	Per cent (%)
Type of study		
Cost to health systems	53	98.1
Cost-benefit analyses	10	18.5
Focus of study		
Elimination	22	40.7
Control	32	59.3
Publication date		
On or after 2000	26	48.1
Before 2000	28	51.9

### Cost to the health system

Among the 53 studies that reported the cost of malaria on health systems, 32 were on the cost of control (Table 2; Additional file 1: Table S1) and 21 on elimination and eradication (Table 3; Additional file 1: Table S1). These studies reported direct costs associated with a entire malaria programme or a set of control and elimination interventions. The earliest study was published in 1903, with about 47% of studies being published on or after 2000. Seven studies looked at the costs of malaria control and elimination during the GMEP era (1955–1969). More than half (27) of the studies were on Asian countries, such as India, Sri Lanka and Thailand, and a number of states in western Asia. Eight studies were in African countries, while another 12 had a global, regional or multi-country focus. Five studies were in South American countries and only one was in Europe. Overall, programmatic costs varied immensely from a few hundred dollars to a several hundred million, owing to heterogeneity in study setting or geographic reach, study period, mix and scale of interventions, and costing methodology, among others. Tables 2 and 3 summarize the findings by country, region, focus (malaria control and elimination), and study period.

**Table 2:** Cost of malaria control to the health system

Country or region	Study period	Cost per capita (2013 US$)^a^	Cost per PAR (2013 US$)	Source
Global	2006–2015	2.50	Not provided	[25]
	2003–2009	Not provided	1.42–11.13	[26]
	2002–2007	Not provided	0.47–0.80	[27]
Africa				
Ethiopia	2011–2015	1.67	2.94	[28]
Kenya	1990	0.28	Not provided	[29]
Liberia	1953–1961	31.25–39.06	Not provided	[30]
Mauritius	10-year time horizon	2.37	2.37	[13]
Rwanda	2011–2015	4.76	6.64	[28]
Senegal	2011–2015	4.26	4.26	[28]
Sub-Saharan Africa	2003	1.21–2.22	1.76–2.61	[31]
	2006–2015	3.47	4.65	[32]
Swaziland	10-year time horizon	0.94	4.88	[13]
Tanzania	2011–2015	2.14–2.21	2.14–2.21	[28]
	10-year time horizon	3.26	3.26	[13]
	2011–2015	2.87	2.87	[28]
Zambia	1929–1949	11.86	Not provided	[33]
Americas				
Brazil	1989–1996	2.15	6.60	[34]
Colombia	1993–1998	0.54–3.48	Not provided	[35]
Asia				
Afghanistan	1953	1.34	Not provided	[36]
Bangladesh	2008–2012	Not provided	0.40	[37]
	1990	Not provided	0.02	[38]
China	10-year time horizon	0.12–0.21	0.16–0.22	[13]
India	1953	0.30	Not provided	[36]
	1990	Not provided	0.12	[38]
	1953–1977	0.36	Not provided	[39]
	1989	9.39	Not provided	[40]
Indonesia	1990	Not provided	2.16	[38]
Nepal	1990	Not provided	0.52	[38]
	Unspecified	0.11–1.21	Not provided	[41]
	1984–1985	0.45–1.36	Not provided	[42]
Palestine	1921–1922	19–32	Not provided	[43]
Sri Lanka	2009	Not provided	1.95	[44]
	2004	Not provided	0.87–2.06	[44]
	1994–1995	Not provided	0.36–4.26 per person protected^c^	[45]
	1977–1981	1.71	Not provided	[46]
	1953	0.80	Not provided	[36]
	1934–1955	0.63–5.22	Not provided	[47]
Thailand	1995	Not provided	12.94–15.40 per case^b^	[48]
	1990	Not provided	1.59	[38]

^a^Unless otherwise stated, the costs reported here are costs per capita, computed by dividing total program costs by the total population in the area of implementation

^b^These costs represent the costs for detecting and treating cases and may not include prevention costs

^c^These costs reflect the cost of selected interventions and not the entire programme

**Table 3:** Cost of malaria elimination to the health system

Country or region	Study period	Cost per capita (2013 US$)^a^	Cost per PAR (2013 US$)	Source
Africa				
Mauritius	10-year time horizon	4.63	4.63	[13]
	1955–2008	3.03–6.22	Not provided	[49]
São Tomé and Principe	2007 (modeled over 20 years)	12	Not provided	[50]
Swaziland	2007 (modeled over 20 years)	3.00	Not provided	[50]
	10-year time horizon	2.65	13.77	[13]
Tanzania	10-year time horizon	4.22	4.22	[13]
Americas				
Mexico	1971–1976	0.18	Not provided	[51]
	1970	0.54	Not provided	[52]
Asia				
China	1994–1995	1.23 per suspected case^b^	0.05	[53]
	2007 (modeled over 20 years)	0.27	2	[50]
	2007 (modeled over 20 years)	0.27	2.17	[54]
	10-year time horizon	0.23–0.54	0.30–0.55	[13]
India	Unspecified	Not provided	0.58 per person protected	[10]
Indonesia	Unspecified	Not provided	0.97 per person protected	[10]
Iran	Unspecified	20.95	Not provided	[55]
Iraq	1964–1970	2.96	Not provided	[56]
Jordan	1964–1970	0.95	Not provided	[56]
Lebanon	1964–1970	1.68	Not provided	[56]
Philippines	1998–2010	Not provided	0.67–13.08	[57]
Solomon Islands	2008	1.60	Not provided	[58]
	2007 (modeled over 20 years)	20	Not provided	[50]
Sri Lanka	2007 (modeled over 20 years)	1.00	Not provided	[50]
	Unspecified	Not provided	0.86 per person protected^c^	[10]
Syria	1964–1970	0.73	Not provided	[56]
Taiwan	Unspecified	Not provided	0.52 per person protected^c^	[10]
	1952–1957	15.06	Not provided	[59]
Thailand	Unspecified	Not provided	1.54 per person protected^c^	[10]
Vanuatu	2008	3.34	Not provided	[58]
	2007 (modeled over 20 years)	27	Not provided	[50]
	1991	18.44	Not provided	[60]

^a^Unless otherwise stated, the costs reported here are costs per capita, computed by dividing total program costs by the total population in the area of implementation

^b^These costs represent the costs for detecting and treating cases and may not include prevention costs

^c^These costs reflect the cost of selected interventions and not the entire programme

### Health system costs of malaria control

Of the 32 studies on costs of malaria control, only 24 (45%) used empirical data such as public and private expenditure reports or survey data. Eight studies used historical expenditures and budgets to extrapolate the costs of intensive control in Africa [28, 31, 32, 61], India [62], Thailand [48], Nepal [41], and globally using varying time periods [63].

**Fig. 2:**
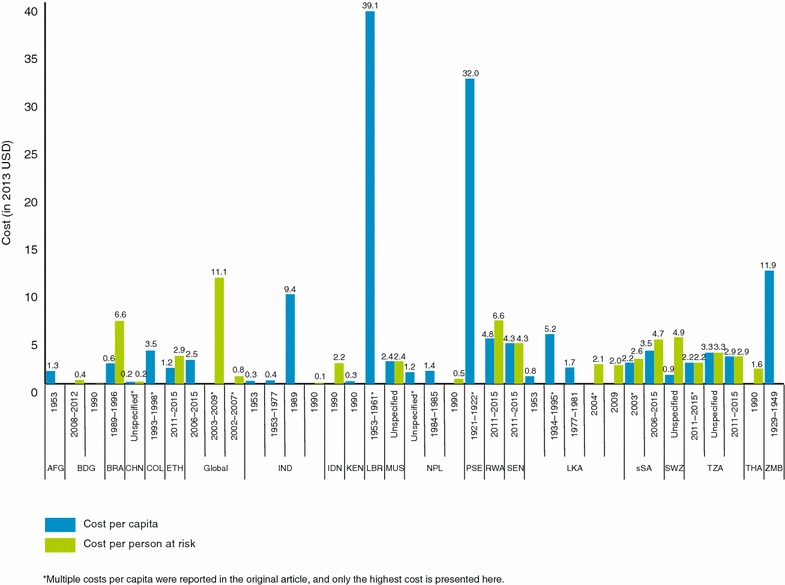
Cost per capita and cost per population at risk of malaria control. *AFG* Afghanistan, *BDG* Bangladesh, *BRA* Brazil, *CHN* China, *COL* Columbia, *ETH* Ethiopia, *IND* India, *IDN* Indonesia, *KEN* Kenya, *LBR* Liberia, *MUS* Mauritius, *NPL* Nepal, *PSE* Palestine, *RWA* Rwanda, *SEN* Senegal, *LKA* Sri Lanka, *sSA* Sub-Saharan Africa, *SWZ* Swaziland, *TZA* Tanzania, *THA* Thailand, *ZMB* Zambia

The median annual cost per capita for malaria control across all studies was $2.21 (range $0.11–$234.17). Sabot et al. (China), Some et al. (Kenya), Ramaiah (India), and Haque et al. (Bangladesh) reported some of the lowest per capita costs at $0.12–$0.21, $0.28, $0.36, and $0.40, respectively (Table 2; Fig. 2) [13, 29, 37, 39]. Two studies by Mills showed comparatively low per capita costs for malaria control in Nepal across several districts, ranging $0.11–$1.36 [41, 42]. Control costs ranged from $0.11 in Nepal [38] to $9.39 in India [40], $32 in Palestine [43] to $39.06 in Liberia [30]. In Nepal and India, the costs included interventions such as testing and treatment, indoor residual spraying (IRS), and bed nets, while in Palestine and Liberia they included community education, environmental management and chemoprophylaxis. Costs also varied within countries over time, partly due to the mix of interventions that were included in the costing. For example, in India, control costs were reported at $0.36–$0.58 during the GMEP era. Costs were generally lower in Asia compared to Africa.

In a sub-set of 13 studies conducted after 2000, of which only ten were conducted in Africa, control costs ranged from $0.94 in Swaziland and $4.75 per capita in Rwanda (median $2.30 per capita). In Asia costs ranged from 0.40 per capita in Bangladesh and $2.06 per capita in Sri Lanka (median $0.64). Most of these studies did not use the full package of WHO recommendations for malaria control at scale. None of the studies in the Americas has been conducted since 2000.

Stuckey et al. [61] modelled the cost of implementing distribution of long-lasting insecticidal nets (LLINs), IRS, and intermittent screening and treatment among school children twice per year at 80–90% coverage in Nyanza Province of western Kenya at $179.50–$234.17 annually per capita. However, these costs were based on modelled coverage of interventions rather than actual scales.

With respect to cost per PAR, the overall median cost per PAR for malaria control, across all studies was $2.15 (range $0.02–$11.13). Kondrashin reported the lowest cost per PAR at $0.02 in Bangladesh, followed by $0.12 in India and $0.52 in Nepal (Fig. 2) [38]. Snow et al. [27] also reported low cost per PAR ($0.47–$0.80) for *Plasmodium falciparum* infections across 87 countries. These two studies used aggregated budget data from WHO, Global Fund and the World Bank. Only two studies that used empirical data reported cost per PAR, which ranged from $0.87 to $1.95 in Sri Lanka [44] and $6.64 in Rwanda [28].

### Health system costs of malaria elimination

Analyses of actual expenditures for programmes that have recently or are currently eliminating malaria have been conducted in only a few selected places, primarily in Asia and Africa with some work in South America and Europe (Table 2; Additional file 1: Table S1). Of the 21 studies on costs of malaria elimination with known data sources, only 11 used empirical data. Eight of the 21 studies looked at the prospective costs of elimination and eradication while the rest used retrospective costs.

**Fig. 3:**
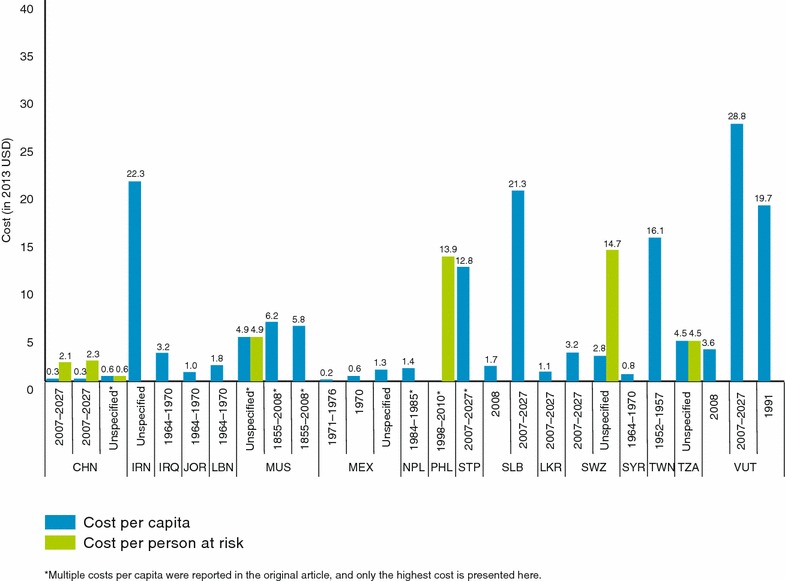
Cost per capita and cost per population at risk of malaria elimination. *CHN* China, *IRN* Iran, *IRQ* Iraq, *JOR* Jordan, *LBN* Lebanon, *MUS* Mauritius, *MEX* Mexico, *NPL* Nepal, *PHL* Philippines, *STP* São Tomé and Principe, *SLB* Solomon Islands, *LKR* Sri Lanka, *SWZ* Swaziland, *SYR* Syria, *TWN* Taiwan, *TZA* Tanzania, *VUT* Vanuatu

Total programmatic costs of malaria elimination ranged from $10,472 in Iran per 500 population (or $20.95 per capita) [55] to $27 million per year in South Africa [64] (or $ 0.52 per capita) (Additional file 1: Table S1). The median annual cost per capita for malaria elimination across all studies was $3.00 (range $0.10–$20.95) In Iran the assumptions for each type of intervention included were not uniform. Larviciding and IRS were implemented annually, however it is unclear if the costs for insecticide-treated nets (ITNs) and treatment were yearly. In terms of cost per capita, the range of reported costs was $0.18 in Mexico in 1971 [51] to $0.27 in China [76], $15.06 in Taiwan [59], $20.95 in Iran [84], and $27 in Vanuatu [50] (Table 2; Fig. 3). A study in the Aneityum Island of Vanuatu reported the second highest cost per capita at $18.44 [60]. This 1991 campaign included weekly mass drug administration (MDA), ITN distribution, and the use of larvivorous fish in breeding sites and was successful in ending local transmission. Barring a few exceptions, reported elimination costs per capita were generally lowest in the Asian countries (i.e., China [13, 50, 53, 54], India [10], Indonesia [10], Philippines [57], Taiwan [10], Thailand [10], Sri Lanka [50], and Vanuatu [58]) and Mexico [51, 52]. Costs were generally highest in African nations, such as Mauritius [49], São Tomé and Principe [50], Swaziland [13, 50], and Tanzania (Zanzibar) [13].

Assessing a sub-set of 12 studies carried out after 2000, five were carried out in Africa, eight in Asia including one carried out in the Philippines between 1998 and 2010 and six with unspecified dates. In the eight studies carried out in the Asia Pacific, costs ranged from $0.27 per capita in China to $27 in Vanuatu (median $1.30). Elimination costs were higher in Africa, with costs ranged from $2.65 in Swaziland to $4.22 in Tanzania and $12 in Sao Tome (median $4.22 per capita).

In terms of cost per PAR, elimination in China has the lowest average annual cost of $0.05 [53] (Table 2; Fig. 3). Similarly, modeled costs per PAR for elimination in China were $0.30 in Jiangsu and $0.50 in Hainan, while POR is estimated to be $0.13 per PAR in both provinces [13]. Other countries report much higher cost per PAR. For example, the cost of Mauritius’s second elimination campaign in 1975–1990 was approximately $4.63 per PAR per year, even though several economic costs and contributions by external partners were not included [9, 49]. Costs per PAR in different provinces in the Philippines ranged from $2.77 to $4.33 (excluding outbreak years) [57]. Four countries in the Middle East reported similar costs per PAR in 1970 ranging from $0.73 to $2.96 [56].

### Economic benefits

Several studies explored the other economic benefits of investing in malaria control and elimination without a cost component. Two studies found that a reduction in malaria burden was associated with increased household spending in India [65] and increased household consumption in Vietnam [66]. In the USA and Latin American countries, exposure to malaria elimination programmes was associated with less work disability [67] and higher incomes [68] in adulthood. In a widely cited study, Gallup et al. [69] found that a 10% reduction in malaria burden was associated with as much as 0.3% in gross domestic product (GDP) growth. Finally, Hong found that between 1850 and 1860 in the USA, people who migrated from one area to another place with less malaria accumulated greater real estate wealth compared to those who relocated to a more malarious area [70].

### Cost-benefit analyses

Of the ten CBAs identified (Table 4; Additional file 2: Table S2), three were conducted during the GMEP era [39, 71, 72] and five were on malaria elimination. Eight were original studies while two articles were reviews with overlapping studies included [10, 15] and only two used empirical data [39, 72]. The main type of economic benefit identified in the studies was increased labour productivity due to reductions in morbidity and absenteeism. Other benefits included reductions in treatment costs and gains from the migration of labour into previously malarial areas.

**Table 4:** Cost-benefit analyses

Country or setting	Study period	Focus (control or elimination)	Benefit-cost ratio	Source	Quality assessment score (out of 10)
Global	2010–2030	Elimination	6.11	[73]	7
Greece	1946–1949	Elimination	17.09^a^	[74]	1
India	1953–1954, 1976–1977	Control	9.27	[39]	6
	2000–2001	Control	4.14^a^	[75]	3
Iraq	1958–1967	Elimination	6.3^a^	[71]	3
Paraguay	1965	Elimination	2.6–3.3	[72]	3
Philippines	Unspecified	Control	2.4	[15]	NA
Sri Lanka	1947–1955	Control	146.3	[15]	NA
Sub-Saharan Africa	Varies by study	Control	1.9–17.1	[10]	NA
Sudan	1977–1984	Control	4.6	[15]	NA
Thailand	Unspecified	Control	6.5	[15]	NA
West Pakistan	1960	Control	4.9	[15]	NA
Zambia	1929–1949	Control	0.57^a^	[33]	9
	2006–2015	Control	40	[28]	6

^a^Calculated by authors based on reported benefits and costs

All but one study in Zambia [33] showed a positive BCR, with BCRs for control ranging from 2.4 in the Philippines [10], 4.14 and 9.22 in India [39, 75] and 17.09 in Greece [74], to over almost 150 for elimination in Sri Lanka [15] (Table 3).

### Quality assessment and critical appraisal

The results of the quality assessment of CBAs using the Drummond ten-point checklist are in Additional file 3: Table S3. Out of a possible ten points, the average score for CBAs was 4.8 (range 0–9). Several CBA studies scored poorly for failing to discount future benefits, identify alternative scenarios and conduct incremental analyses, carry out sensitivity analyses, and address key issues related to resource allocation in the country or setting where the study was situated. Additional file 4: Table S4 shows the results of quality evaluation of the cost studies. Over half of studies that evaluated programmatic costs described their cost inputs and thus scored high on the scope of costing metric of the Fukuda and Imanaka criteria. Although a total of 54 studies were evaluated in this review, strong conclusions cannot be drawn and the findings should be interpreted with caution.

## Discussion

Summarizing evidence on economics of malaria from heterogeneous studies, sources, inputs, methods, time, and geography is challenging. While total costs were corrected for population size by presenting them as cost per capita or cost per PAR, other factors contributed to the magnitude of the costs. The methodologies and cost inputs used were not standard and many studies used secondary data. In some cases, the cost inputs, cost categories, interventions, and assumptions that were included were not stated explicitly. Some studies provided coverage inputs, such as total population or PAR, while others presented a simple total programmatic cost. Among the studies included in the review, discount rates when specified, ranged from 3 to 16%. Many of the studies included used a public sector perspective for economic analysis. However, these costs represent only part of the equation. While most malaria control efforts are largely government-led public health initiatives, programmatic costs are only part of the picture as individuals, households and employers from the private sector may also incur costs for malaria treatment and prevention. It is unclear to what extent these direct and indirect costs were included in the literature examined. Out-of-pocket expenditures for treatment as well as transport to health facilities, as well as any indirect opportunity cost of lost wages and absenteeism, may have substantial consequences. Other studies have shown that up to 6% of a household’s total spending on health, even when public sector primary health care is free and indirect costs can translate to $150 in lost earnings per malaria episode [76].

Numerous caveats with respect to the relevance and extrapolation of the results exist and findings should be used cautiously. First, programme costs depend largely on the mix and scale of interventions, which differ from country to country, or even among districts or provinces in countries with decentralized systems. Mauritius, for example, employs a more costly border-screening programme for visitors from malaria-endemic countries. Some earlier studies did not incorporate post-elimination costs of surveillance and other interventions to prevent re-introduction of the disease, as the expectation at the time was that malaria-related expenditure would stop after elimination. In the early studies that did actually demonstrate reductions in post-elimination expenditures, the value of these savings were diminished due to discounting, preventing them from fully offsetting the initial increased investments to reach elimination. Second, cost is affected by the size and programme efficiency of a health system used to implement interventions, as well as the coverage rates employed. Smaller countries such as Swaziland potentially due diseconomies of scale appear to have higher costs. Sri Lanka on the other hand, has one of the earliest and effective public health systems with generally low levels of health expenditures. Costs also differed by the region (Africa or Asia) with costs in Asia much lower than in Africa, possibly due to higher use of vector control in Africa, as well as size and development status of the country evaluated. Fourth, timing plays an important role in determining the price of consumables, services and labour. Estimates from earlier years were generally lower than that from the contemporary studies due to the difference between the relative prices of physical and human inputs to malaria control. In addition, the current menu of tools and interventions for malaria is broader and more costly, encompassing LLINs, intermittent preventive therapy for pregnant women and children, artemisinin-combination therapy, and rapid diagnostic tests, as well as innovative delivery models. Lastly, there are wide variations in regional, epidemiological and economic contexts. The presence of the more tenacious *Plasmodium vivax* could have substantial cost implications during the elimination phase. Barring a few studies based on mathematical models, few measured the cost of the full spectrum of WHO recommendations for the control of malaria. For elimination, there is currently no recommended optimal package as the interventions are often context specific and tailored to the particular landscape of the country. While some of these programmatic, temporal, spatial, and methodological differences are expected in costing studies, future studies should attempt to standardize methodologies to facilitate meaningful comparisons of cost estimates.

Despite the challenges in directly comparing costs in the studies reviewed, some trends can be observed. While the investment needed to achieve elimination varied greatly between countries and contexts, it is likely that the immediate costs for elimination will initially be equal to, or higher than those of a control programme, as indicated by data from Swaziland [13], due to initial investments in programme re-orientation to strengthen surveillance systems. This cost however tend to decrease as the focus progresses to the POR phase [42–44] due to streamlining of surveillance activities, reductions in commodity expenditures and in some cases, integration of supporting health system activities [13, 77]. Two studies that collected empirical data on actual expenditures over multiple programmatic phases support this claim. In Sri Lanka, expenditures per PAR declined when moving from a high level of control to controlled low-endemic malaria [44]. In the Philippines declining marginal expenditures were observed from control to POR, where costs per PAR were more than halved [57]. Similar findings have been reported in three Namibian regions in a recent study published after the initial search was conducted [78]. In contrast, Ruberu’s analysis in Sri Lanka suggested that the high short-term cost of elimination is exceeded by long-term investments in control and the resulting consequences of productivity losses [46]. This is supported by the Eighth Report of the Expert Committee on Malaria which suggested that the cost of a well-operated programme to consolidate and sustain elimination would be only 65–75% that of operating an ‘all-out’ or intensive malaria control programme [79].

The bulk of the CBAs dated from the GMEP era. Several of these studies focused on periods of relatively high transmission (i.e., control), even though elimination or eradication was mentioned in the title or body of articles, emphasizing the need to standardize the use of malaria terminology. Most of these studies were prospective in design and suggest that the benefits of intensive control and elimination exceed costs. However, these studies have not been followed up subsequently to assess the validity of their conclusions. The main type of economic benefit identified in the studies was increased labour productivity due to reductions in morbidity and absenteeism. Other benefits included reductions in treatment costs and gains from the migration of labour into previously malarial areas. Factors such as school absenteeism due to malaria and its effect on cognitive development and educational outcomes have also been reported by several studies, for example, Lucas reported that in Sri Lanka ending malaria in the most heavily affected region led to an estimated 17% increase in literacy [80]. Similarly, Bleakley et al. [81] examined the effects of malaria on female educational attainment in Paraguay and found that every 10% decrease in malaria incidence led to 0.1 years of additional schooling, and increased the chance of being literate by one to two percentage points. While an important factor on human capital accumulation, these were not included in this review as they did not present costs in economic terms, an important element in order to be comparable and used in economic analyses.

As with cost estimates, the heterogeneity in cost-benefit estimates can be explained largely by the lack of standardization in calculating BCRs, particularly on how benefits were defined, categorized or estimated. Some studies used a broad definition of benefits from a societal perspective, while others used a narrow definition of outputs. Some studies also made wide-ranging assumptions about the effect of malaria on labour, tourism and the larger economy and attempted to include their effect into their metric. The studies also use varying time periods of analysis and a variety of discount rates ranging from 3 to 10% to obtain present values. A complete economic assessment of elimination should include direct and indirect benefits, some of which are difficult to measure. The economics of malaria elimination are complicated because most of the benefits of elimination are typically realized only when an absolute threshold of malaria-free status is achieved, by conferring indirect benefits such as economic development [82]. While it is expected that one of the benefits of malaria is likely to be a positive effect on tourism, two studies carried out in an area of South Africa and Mauritius [83, 84] reported that tourists’ perceptions of risk were highly unresponsive to actual changes in malaria transmission. A comprehensive CBA should compare the potential net benefits of elimination with those of control. Ideally, such as exercise should begin with cost-minimization analysis to establish the optimum package of interventions with which to achieve control and elimination. Nevertheless, the overall favourable BCR of investing in malaria supports the case for continued investment in malaria elimination within individual countries and globally.

Few studies have looked at the relative returns to elimination *versus* long-term control. The Eighth Report of the Expert Committee on Malaria (1961) suggested that experience indicated that a well-operated consolidation mechanism costs per annum 65–75% of an attack mechanism [79], and there is some evidence that the costs for elimination are likely to be equal to or higher than those of a control programme [50, 85]. Indeed, one of the strongest arguments against elimination is the increasing cost associated with finding and treating decreasing numbers of cases, since the final few cases require an enormous outlay of resources that may be considered disproportionate to the marginal return [86]. This discussion around the financing of malaria elimination is no different to that of other elimination and eradications programmes. Since the start of the Global Polio Eradication Initiative (GPEI), the burden has been reduced by over 99%. Twenty-seven cases of wild polio have been diagnosed this year, all in Afghanistan, Pakistan and Nigeria. Finishing the job of eradicating polio will cost an additional $1.5 billion to enhance vaccination and surveillance efforts in hard-to-reach places. This translates into a cost of about $0.5 billion a year or $18 million per case averted. However, eradicating polio will have saved at least $40–50 billion between 1988 and 2035. In the USA alone, eradicating polio is estimated to have saved about $220 billion since 1955. Nevertheless, some public health advocates continue to question whether polio should be merely managed rather than eliminated and the money be allocated to fighting other diseases. However, withdrawing support will have devastating health, social and economic effects. In 2003, certain states in Nigeria briefly stopped delivering vaccines in 2003 and as a result, GPEI spent $220 million dealing with the resultant outbreak. Equatorial Guinea also recently saw its first reported polio case since 1999, when a virus from Cameroon exploited a drop in the routine vaccination of children [87, 88].

Similarly, while the literature supports the claim that investment in malaria elimination provides generous benefits, the challenge is sustaining financial support. Donor funding is on the decline in favour of programmes with seemingly greater potential impact on mortality and morbidly. Although many of the countries currently attempting to eliminate malaria are middle-income countries and will eventually be able to fund their programmes domestically, they are faced with competing priorities for finite amounts of financing. In addition, the long-term nature of elimination programmes contrasts with governments’ and donors’ typical short-term funding cycles and goals. As a result, elimination programmes become victims of their own success and risk the withdrawal of funding at a critical time in their malaria epidemiology.

The review identified several gaps in the literature on the economics of malaria elimination. Firstly, there is no standard methodology or guidance for computing the cost of malaria control and elimination. The studies in this review employed a wide range of inputs to compute the cost of malaria control and elimination to arrive at the costs, making meaningful comparisons difficult. For elimination, this standardization needs to include the cost likely to be incurred in a post-elimination scenario to allow appropriate budgeting and planning. Secondly, while comprehensive WHO guidance exists on interventions for the control of malaria, there is little direction on the epidemiological and economic efficiencies of various mixes of interventions utilized for malaria elimination. The start-up costs of malaria elimination, particularly the cost of strengthening surveillance systems for enhanced case identification are also largely unknown. A country embarking on elimination will need to plan for the additional resources needed in its transition from control to elimination. Most of the studies in this review used financial costs and therefore, the true cost of the human resources and programmatic management and health system strengthening are largely unknown. Lastly, malaria elimination confers several non-health benefits to the economy. Methods to comprehensively quantify these benefits will greatly enable stakeholders to strengthen the elimination argument.

## Conclusion

The evidence documented in this review is important in answering key questions on resource allocation and financial planning by malaria programme managers and policymakers serving as an interim guide for countries until they are able to undertake more robust economic analyses in their own contexts. The investment needed to achieve elimination is likely to initially be equal to or higher than that of a control programme, particularly in the short term. As with any disease elimination programme, the cost of ‘finishing the job’ is likely to be higher than merely controlling the disease. This higher cost must be built into programme budgets with appropriate advocacy actions to ensure that financing is maintained well after elimination is achieved. At the same time, it should be tacit that, the total benefits of elimination, many immeasurable, vastly outweigh its cost. Nevertheless, there is a need for thorough research into the comprehensive benefits of elimination to guide relevant policy decisions. At the same time, malaria-related expenditure is not likely to stop as soon as elimination is achieved. Malaria interventions need to be viewed as a continuous expenditure even when the disease is absent, such as with routine immunization, until global eradication is achieved. Elucidating the health and economic costs and comprehensive benefits of continuing spending will facilitate such a policy shift.

## Additional files

**Additional file 1: Table S1.** Cost of malaria to the health system.

**Additional file 2: Table S2.** Cost-benefit analyses of malaria control and elimination.

**Additional file 3: Table S3.** Quality assessment of CBAs.

**Additional file 4: Table S4.** Quality assessment of costing analyses.
