# Supplementary material for: The economics of malaria control and elimination: a systematic review
Source: Malar J. 2016 Dec 12;15:593. doi: 10.1186/s12936-016-1635-5 (PMC5154116; doi:10.1186/s12936-016-1635-5)
Supplement: Supplementary file 1 — Additional file 1: Table S1. Cost of malaria to the health system. [file 12936_2016_1635_MOESM1_ESM.docx]

**S1 Table. Cost of malaria to the health system**

| **Source**^[[1]](#footnote-1)^ | **Country or region** | **Study type/**  **Study method** | **Study period** | **Data source** | **Costs and interventions included** | **Total cost of program (2013 US$)**^[[2]](#footnote-2)^ | **Cost per capita per year (2013 US$)**^[[3]](#footnote-3)^ | **Cost per PAR per year (2013 US$)**^[[4]](#footnote-4)^ |
| --- | --- | --- | --- | --- | --- | --- | --- | --- |
| Abeyasinghe et al. (2012) [1] | Sri Lanka (Kurunegala and Anuradhapura districts) | Retrospective/  Cost analysis | 2004 and 2009 | Literature search, public sector expenditure records, informant interviews | Prevention, diagnosis, treatment and prophylaxis, surveillance and response, education and communication, and program management | No total cost provided | No total population provided | Anuradhapura: 0.87 (2004) and 1.95 (2009)  Kurunegala: 2.06 (2004) and 1.95 (2009) |
| Akhavan et al. (1999) [2] | Brazil (Amazon basin) | Retrospective/  Cost analysis and CEA | 1989-1996 | Literature for epidemiological data, unclear for cost data | Prevention and treatment | 914 M (780 M prevention, 134 M treatment) | 2.57 (2.18 prevention, 0.38 treatment)5 | 2.57 (2.18 prevention, 0.38 treatment)^[[5]](#footnote-5)^ |
| Clinton Health Access Initiative, et al. (2011) [3] | Ethiopia, Rwanda, Zambia, Tanzania (Mainland and Zanzibar) | Prospective/  Cost analysis and CEA | 2011-2015 | Malaria specific expenditures from government and active partners | Diagnosis and treatment | Ethiopia: 148 M  Rwanda: 55 M  Senegal: 55.4 M  Mainland Tanzania: 88-91 M  Zanzibar: 4 M | Ethiopia: 1.675  Rwanda: 4.785  Senegal: 4.265  Mainland Tanzania: 2.14-2.215  Zanzibar: 2.875 | Ethiopia: 2.945  Rwanda: 6.645  Senegal: 4.265  Mainland Tanzania: 2.14-2.215  Zanzibar: 2.875 |
| Dua et al. (1997) [4] | India (one industrial setting) | Prospective and retrospective/  Cost analysis | 1987-1995 | Entomological and parasitological surveys, hospital budgets | Direct cost to health facilities | 112,000 (1985)  684,000 (1986-1995) | No total population provided | No PAR provided |
| Dy (1954) [5] | Various countries in Asia | Retrospective/  Cost analysis | 1953 | Public sector expenditure records | Personnel, supplies, equipment, transport, and other miscellaneous expenses | Afghanistan: 726,000 Burma: 284,000  Ceylon: 6.1 M  China: 205,000  India: 10.9 M  Indonesia: 160,401  Malaya: 24,900  Portuguese India (Goa): 64,700  Thailand: 1.8 M  Vietnam: 3.2 M | Afghanistan: 1.34  Ceylon: 0.80  India: 0.30  *Cost per person protected* Afghanistan: 1.74  Burma: 2.74  Ceylon: 1.98  China: 1.37  India: 0.61 Indonesia: 1.88  Malaya: 5.80  Portuguese India (Goa): 2.32  Philippines: 4.25  Thailand: 9.71  Vietnam: 1.06 | No PAR provided |
| Ebi (2008) [6] | Global | Prospective/  Cost analysis | 2000-2030 | WHO database, Disease Control Priorities II project cost data | ITNs, case management with ACT, IPTp, and IRS | 1.701 M-9.503 M^[[6]](#footnote-6)^ | No total population provided | No PAR provided |
| Giron et al. (2006) [7] | Colombia | Retrospective/  Cost analysis and CEA | 1993-1998 | Public sector expenditure records, household interviews | Fumigation, spraying, bednet treatment, elimination of breeding sites, IEC on environmental factors, and malaria tests | National program: 5,380 per 10,000 persons  Integrated alternative: 34,847 per 10,000 persons | National program: 0.54  Integrated alternative: 3.48 | No PAR provided |
| Gunaratna (1956) [8] | Ceylon (Sri Lanka) | Retrospective/  Cost analysis | 1934-1955 | Unspecified | Spraying, case detection, and treatment | 98,000-7.3 M | 0.63-5.22 | No PAR provided |
| Haque et al. (2014) [9] | Bangladesh | Retrospective/  Cost analysis | 2008-2012 | Public sector expenditure record | Equipment, infrastructure, training, operational research, transportation, and supplies such as drugs, diagnostics, LLINs, and insecticides for retreatment of nets | No total cost provided | 0.40 | No PAR provided |
| Hedman et al. (1979) [10] | Liberia (Yekepa, Nimba County) | Retrospective/  Cost analysis | 1953-1961 | Unspecified | Vector control measures (including personnel, chemicals, equipment) and chemoprophylaxis with amodiaquine | 504,969 | 31.25-39.06 | No PAR provided |
| James (1903) [11] | India (Mian Mir cantonment) | Retrospective/  Cost analysis | 1901-1903 | Unspecified | Personnel, environmental management for vector control, and miscellaneous expenses | 7,217 rupees^[[7]](#footnote-7)^ (1901-1902) | 4.70 rupees11 | No PAR provided |
| Jowett et al. (2005)** [12] | Tanzania | Retrospective/  Cost analysis | 1998 | Literature, donor and public sector expenditure records, manufacturer’s pricing for drug prices | Prevention and treatment activities | 93 M | 3.14 (government 0.63, donors 0.30, private 2.21) | No PAR provided |
| Kaewsonthi et al. (1989) [13] | Thailand | Unclear/  Cost analysis | Unspecified | Unspecified | Surveillance, vector control, and malaria clinics | 123 M (24.3 M government, 98.7 M private) | No total population provided | No PAR provided |
| Kamolratanakul et al. (1999) [14] | Thailand | Prospective/  Costs analysis | 1995 | Unspecified | Personnel, materials, and capital | 88,737 | Cost per *Pv* case: 12.94  Cost per *Pf* case: 15.40  Cost per visit: 2.59, Cost per case: 11.48  Cost per house sprayed: 3.13  Cost per impregnated net: 2.15 | No PAR provided |
| Kiszewski et al. (2007)** [15] | 81 high-burden malaria countries | Retrospective/  Cost analysis | 2006-2015 | WHO database, UNDP projections, public sector expenditure record | Commodities and distribution, health system strengthening activities, training, communication, operational research, M&E, and technical assistance | 4.4 B-5.2 B per year (2 B-2.5 B Africa, 2.4 B-2.8 B rest of the world) | Africa: 2.81  Asia and Oceania: 1.34  Americas: 0.99  Global: 2.50 | No PAR provided |
| Kligler (1924) [16] | Palestine | Retrospective/  Cost analysis | 1921-1922 | Unspecified | Case detection and treatment, vector control, prophylaxis, and education | Migdal: 434  Kinnereth: 677  Yemma: 812 | Migdal: 24  Kinnereth: 32  Yemma: 22  Menachamia: 19  Um-Ul-Alex: 32 | No PAR provided |
| Kondrashin (1992) [17] | WHO SEARO region | Retrospective/  Cost analysis | 1990 | WHO SEARO and New Delhi budget data | Unspecified | No total cost provided | No total population provided | Bangladesh: 0.02  India: 0.12  Indonesia: 2.16  Nepal: 0.52  Thailand: 1.59 |
| Konradsen et al. (1999) [18] | Sri Lanka (one area in Anuradhapura district) | Retrospective/  Cost analysis | 1994-1995 | MOH, Anti Malaria Campaign, Kekirawa government hospital, survey data | Salaries, transport and storage, chemicals, capital investments and maintenance for IRS, bednet impregnation, larviciding, water management, and diagnosis and treatment | No total cost provided | *Cost per person protected per year*  Spraying: 3.13-4.26  Bednet impregnation: 1.29  Larviciding: 0.73  Water management: 0.36  *Cost per positive case*  Diagnosis: 1.45-2.39  Treatment: 1.91-4.12 | No PAR provided |
| Korenromp et al. (2013)* [19] | 90 countries | Retrospective/  Cost analysis | 2003-2009 | Disbursement reports from donors, WHO database, household surveys, manufacturer cost reports | Unspecified | No total cost provided | 78-5,749 per case prevented^[[8]](#footnote-8)^  57,654-3,903,107 per death prevented8 | 1.42-11.135 |
| Mills (1992)* [20] | Nepal (5 districts) | Prospective/  Cost analysis and CEA | Unspecified | Surveys, government control program | Diagnosis and prevention | No total cost provided | 0.11-1.21 | No PAR provided |
| Mills (1993b) [21] | Nepal | Retrospective/  Cost analysis and CEA | 1984-1985 | Survey data, malaria program budgets and accounts, surveillance data | NMCP costs | Morang: 174,877 and 112,567^[[9]](#footnote-9)^ Ilam: 57,938 and 31,134  Rupandehi: 186,546 and 139,037 | Morang: 0.45 and 0.97  Ilam: 1.35 and 1.36  Rupandehi: 0.81 and 0.875 | No PAR provided |
| Morel et al. (2005) [22] | Sub-Saharan Africa | Prospective/  CEA | 2003 population data as baseline, modeled over 10 years | Literature review, expert opinion, WHO-CHOICE database | Unspecified | Southern and Eastern Africa: 597,045,946-598,568,437  Western Africa: 426,990,689-632,846,172 | Southern and Eastern Africa:  2.22  Western Africa:  1.21-1.80 | Southern and Eastern Africa:  2.26-2.27  Western Africa:  1.76-2.61 |
| Prakash et al. (2003)** [23] | India (Jorajan camp of Oil India, upper Assam) | Retrospective/  Cost analysis and CBA | April 2000-May 2001 | Oil India Limited records | Personnel, transportation, and antimalarial measures | 2,746 | Cost of hospitalization per case: 264.89 | No PAR provided |
| Ramaiah (1980) [24] | India | Retrospective/  Cost analysis and CBA | 1953-1977 | Literature, public sector expenditure reports | Treatment and transportation | 4.274 M | 0.365 | No PAR provided |
| Ruberu (1977) [25] | Sri Lanka | Retrospective/  Cost analysis | 1977-1981 | Malaria program expenditures and reports, source of historical epidemiological data unclear | NMCP costs | 7.2 M-13.2 M (1977-1986)  Attack phase (1977-1981): 120.5 M | Attack phase: 1.71 | No PAR provided |
| Sharma (1996) [26] | India | Retrospective/  Cost analysis | 1991 | Literature, public sector expenditure reports | NMCP expenditures, transportation, personal protection methods, and treatment | 330,464,252-542,423,009 | No total population provided | No PAR provided |
| Snow et al. (2008)* [27] | 87 countries | Retrospective/  Cost analysis | 2002-2007 | GFATM, WHO, World Bank, unilateral and bilateral organizations | Approved fund distributions | 1,114,044,944 | No total population provided | Any risk for *Pf*: 0.47  Stable risk for *Pf*: 0.80 |
| Some (1994)** [28] | Kenya (Uasin Gishu district) | Retrospective/  Cost analysis | Jan-Sep 1990 | Hospital record, absenteeism data from 6 primary schools, routine and verbal reports | Accommodations, vehicle use and maintenance, supplies, printing, equipment and maintenance, and miscellaneous expenses | Additional cost of controlling the malaria epidemic (June 1990): 142,665 | 0.285 | No PAR provided |
| Stuckey et al. (2014) [29] | Kenya (Rachuonyo South district, Homa Bay county, Nyanza province) | Prospective and retrospective/  Cost analysis and CEA | 2011-2012 data as baseline, modeled over 5 years | GFATM, WHO-CHOICE, and Malaria Transmission Consortium databases, literature review, demographic and health survey | Health system resources, treatment, supplies, personnel, and direct patient costs (travel and consumables) | 89,749,493-117,078,093 | 897.49-1170.78 over five years (179.50-234.17 per year^5^) | No PAR provided |
| Teklehaimanot et al. (2007)** [30] | Africa | Prospective/  Cost analysis | 2006-2015 | Literature, UNDP database, UN data on malaria | Prevention, diagnosis, treatment, M&E, and overhead | 3.5 B | 3.47 | 4.65 |
| Utzinger et al. (2002) [31] | Zambia (four communities) | Retrospective/  Cost analysis | 1929-1949 | Census data, life tables, literature search, program budgets for control | Prevention, diagnosis, and treatment | 17,078,703 | 11.865 | No PAR provided |
| Yadav et al. (1991) [32] | India (two mining settlements in Orissa) | Retrospective/  Cost analysis | May 1989 | Hospital records, survey, expenditure data from mining companies | Treatment, antilarvals, and IRS | 128,109 | 9.395 | No PAR provided |
| Beaver (2011) [33] | Solomon Islands, Vanuatu | Retrospective/  Cost analysis | 2008 | Government budget projection reports, GFATM, AusAID, WHO, and Rotary Against Malaria data | Projected budgets for case management, diagnosis, prevention, and M&E | No total cost provided | Vanuatu: 1.60  Solomon Islands: 3.34^[[10]](#footnote-10)^ | No PAR provided |
| Cohn (1973) [34] | India | Retrospective/  Cost analysis | 1952-1971 | National malaria program expenditure data | Materials, equipment, and operations | Control (1951-1958): 150 M  Elimination (1958-1971): 1.3 B | No total population provided | No PAR provided |
| de Zulueta et al. (1972) [35] | Iraq, Lebanon, Syria, Jordan | Retrospective/  Cost analysis | 1964-1970 | Unspecified | NMCP costs | Iraq: 77,083,000 Jordan: 17,699,000 Lebanon: 5,174,000 Syria: 22,067,000 | No total population provided | Iraq (1970): 2.96  Jordan (1970): 1.68 Lebanon (1970): 0.73 Syria (1970): 0.95 |
| Jackson et al. (2002) [36] | China (Gushi and Shangcheng, in Henan province) | Prospective/  Cost analysis | 1994-1995 | Budget for administrative costs, community costs based on sample of suspected cases, government health records | Vector surveillance, population blood surveys, case management, personnel, administration, training, drugs, blood testing, and miscellaneous expenses | 175,340 | 1.23 per suspected case | 0.05 |
| Kahn et al. (2009a) [37] | China (Jiangsu, and Hainan Island), Sao Tome and Principe, Solomon Islands, Sri Lanka, Swaziland, Vanuatu | Prospective/  Cost analysis and CEA | 2007 (modeled over 20 years) | Public sector expenditure reports and budgets, GFATM proposals, expert opinions | NMCP costs | *Jiangsu, China*  Control: 9.9 M  Elimination: 6.66 M *Hainan, China* Control: 3.2 M  Elimination: 2.6 M *Swaziland*  Control: 0.8 M  Elimination 1.36 M | Using GMAP figures (1950s-1960s): 3-14  Hainan, China: 0.27  Sao Tome and Principe: 12  Solomon Islands: 20  Vanuatu: 27  Sri Lanka: 1 Swaziland: 3 | Hainan, China: 2  Sri Lanka: 5  Swaziland: 8 |
| Kahn et al. (2009b) [38] | China (Jiangsu, and Hainan Island), Swaziland | Prospective/  Cost analysis and CEA | 2007 (modeled over 20 years) | China: MOH expenditures and budgets, GFATM proposals, expert opinion Swaziland: government budgets and GFATM proposals | NMCP costs | *Jiangsu, China*  Control: 9.9 M  Elimination: 6.66 M  *Hainan, China* Control: 3.2 M  *Swaziland*  Annual cost: 430,000 Budgeted amount for elimination: 2.6 M | *Hainan, China*  Elimination: 0.27 | *Hainan, China*  Elimination: 2.17 |
| Kaneko et al. (2000)* [39] | Vanuatu (Aneityum) | Retrospective/  Cost analysis | Sept-Nov 1991 | Unspecified | ITNs, antimalarials, microscopy, transportation, and travel allowances | No total cost provided | 18.44 | No PAR provided |
| Liu et al. (2013) [40] | Philippines (4 provinces) | Retrospective/  Cost analysis | 1998-2010 (varies by province) | Subnational historical records, key interviews, Public sector expenditure reports | Diagnosis, treatment, prevention, surveillance, and M&E | Apayao: 384,737-798,470  Laguna: 29,748-117,621 Cavite: 7,464-45,389  Benguet: 17,020-17, 292 | No total population provided | Apayao: 3.50-7.70  Laguna: 3.48-13.08  Cavite: 0.67-4.63  Benguet: 2.69-2.96 |
| Livadas et al. (1963) [41] | Greece | Retrospective/  Cost analysis | 1946-1949 | Unspecified | Direct and indirect cost | 11 M | No total population provided | No PAR provided |
| Lok (1979)* [42] | Singapore | Retrospective/  Cost analyses | 1974-1978 | Unspecified | Program implementation, drugs, and medical care | 3.5 M | No total population provided | No PAR provided |
| Mills (2008) [43] | Multiple countries | Retrospective and prospective/  Cost analysis | Varies by country | Literature | Various | No total cost provided | *Cost per person protected*^[[11]](#footnote-11)^  Taiwan: 0.52  India: 0.58  Sri Lanka: 0.86  Indonesia: 0.97  Thailand: 1.54 | No PAR provided |
| Moonasar et al. (2013) [44] | South Africa | Prospective/  Cost analysis | 2012-2018 | Public sector expenditure reports and budgets | Surveillance, vector control, health promotion, case management, and program management | 190 M (2012-2018) | No total population provided | No PAR provided |
| Niazi (1969) [45] | Iraq | Retrospective/  Cost analysis and CBA | 1958-1967 | Unspecified | Treatment and medical care, antilarval measures, and insecticidal spraying | 86,653,366 | No total population provided | No PAR provided |
| Ortiz (1968) [46] | Paraguay (agricultural, cattle farming, and forestry industries) | Retrospective/  Cost analysis and CBA | 1965 | Servicio Nacional de Erradicación del Paludismo | NMCP costs | Actual value: 38,414,815  Annual disbursement: 51,466,667 | No total population provided | No PAR provided |
| Purdy et al. (2013) [47] | WHO regions | Prospective/  Cost analysis and CBA | 2013-2035 | GMAP | GMAP costs | 7.534 M (2010)  7.163 M (2015)  6.338 M (2020)  6.036 M (2025)  4.167 M (2030)  2.877 M (2035) | No total population provided | No PAR provided |
| Rezaei-Hemami et al. (2014)* [48] | Iran | Retrospective/  Cost analysis and CEA | Unspecified (pre-elimination to elimination phases) | Iranian Ministry of Health and Medical Education | Utilities, capital, operations, personnel, and transportation | 10,472 | 20.95 | No PAR provided |
| Sabot et al. (2010) [49] | China (Hainan and Jiangsu), Mauritius, Swaziland, and Tanzania (Zanzibar) | Retrospective and prospective/  Cost analysis | Varies by country (10-year time horizon for elimination plus 15 years post-elimination) | Public sector expenditure reports and annual health reports, yearly country program data, national health accounts, donor proposals, informant interviews | NMCP costs | *Hainan, China*: Control (2007-2009): 1.766 M  Elimination (2010-2014): 4.72 M  POR (2020-2029): 1.197 M  *Jiangsu, China*  Control (2007-2009): 9.169 M  Elimination (2010-2014): 17.966 M  POR (2020-2029): 8.218 M  *Mauritius*  Control (1982): 2.673 M  Elimination (1983-1988): 4.71 M  POR (1990-2008): 2.999 M *Swaziland*  Control (2004-2008): 1.068 M  Elimination (2009-2013): 3.22 M  POR (2020-2029): 2.452 M *Tanzania*:  Control (2009) 4.229 M  Elimination (2010-2019): 5.31 M  POR (2020-2029): 4.220 M | *Hainan, China*  Control: 0.21  Elimination: 0.54  POR: 0.13 *Jiangsu, China*  Control: 0.12  Elimination: 0.23  POR: 0.10 *Mauritius*  Control: 2.37  Elimination: 4.63  POR: 2.62  *Swaziland*  Control: 0.94 Elimination: 2.65  POR: 1.67  *Tanzania*  Control: 3.26 Elimination: 4.22  POR: 2.18 | *Hainan, China*  Control: 0.22  Elimination: 0.55  POR: 0.13  *Jiangsu, China*:  Control 0.16  Elimination 0.30  POR: 0.13  *Mauritius*  Control: 2.37  Elimination: 4.63  POR: 4.63  *Swaziland*  Control: 4.88  Elimination: 13.77  POR: 8.65  *Tanzania*  Control: 3.26  Elimination: 4.22  POR: 2.18 |
| Suarez Torres (1970a)** [50] | Mexico | Prospective/  Cost analysis | 1971-1976 | Unspecified | IRS, surveillance, case investigation and management, education campaign, entomological surveillance, research, program management, public relations, logistics, and administration | National plan (1971): 856,874  National plan with regional expansion (1971): 1,578,216 National plan with implementation in all malarious areas (1971): 4,057,006  Six-year plan: 21,608,204 | Cost of national plan with implementation in all malarious areas (1971): 0.18 | No PAR provided |
| Suarez Torres (1970b) [51] | Mexico (Gulf of Mexico, Yucatan Peninsula) | Prospective/  Cost analysis | July to Dec 1970 | National Commission for the Eradication of Malaria and federal government | Personnel, supplies, communication, transportation, maintenance, spraying, and vehicles | 537,425 | 0.54 | No PAR provided |
| Taiwan Provincial Malaria Research Institute et al. (1958)** [52] | Taiwan | Retrospective/  Cost analysis | 1952-1957 | Taiwan Provincial Malaria Research Institute | NMCP costs | Total funds for malaria (1952-1956) ^[[12]](#footnote-12)^: 242,705,049 | 15.065 (1956) | No PAR provided |
| Tatarsky et al. (2011) [53] | Mauritius | Retrospective/  Cost analysis | 1855-2008 | Peer-reviewed literature, WHO and government reports, gray literature, expert interviews, budgets, technical reports, program reviews, expenditure data | Surveillance, diagnosis, treatment, prevention, and program management | First elimination (1948-1951): 2.3 M-2.7 M First POR program (1969-1974): 2 M  Second elimination (1982-1991): 3 M-5.6M Current program (2008): 2.7M | First elimination: 4.83 and 6.22  First POR: 3.24  Second elimination: 3.03-5.83 Current POR: 2.23 | No PAR provided |

**Note:** The color scheme in the table represents the focus of each study, where intensive malaria control is white and malaria elimination and eradication are in grey.

**Acronyms used in this table**:

ACT – Artemisinin combination therapy

API – annual parasite index

AusAID – Australian Agency for International Development (now under the Department of Foreign Affairs and Trade)

B – Billion

CBA – Cost-benefit analysis

CEA – Cost-effectiveness analysis

IEC – Information, education and communication

IPTp – Intermittent preventive treatment in pregnancy

IRS – Indoor residual spraying

ITN – Insecticide-treated bednet

GFATM – Global Fund to Fight AIDS, Tuberculosis and Malaria

GMAP – Global Malaria Action Plan

LLIN – Long-lasting insecticidal bednet

M – Million

M&E – Monitoring and evaluation

MOH – Ministry of Health

NMCP – National malaria control program

PAR – population at risk

*Pf* – *Plasmodium facliparum*

POR – Prevention of reintroduction

*Pv* – *Plasmodium vivax*

SEARO – Southeast Asia Regional Office

TWD – New Taiwan dollars

UN – United Nations

UNDP – United Nations Development Programme

WHO – World Health Organization

WHO-CHOICE – WHO cost-effectiveness and strategic planning

**References**:

1. Abeyasinghe RR, Galappaththy GN, Smith Gueye C, Kahn JG, Feachem RG. Malaria control and elimination in Sri Lanka: documenting progress and success factors in a conflict setting. PLoS One. 2012;7(8):e43162.

2. Akhavan D, Musgrove P, Abrantes A, d AGR. Cost-effective malaria control in Brazil: cost-effectiveness of a malaria control program in the Amazon Basin of Brazil, 1988-1996. Soc Sci Med. 1999;49(10):1385-99.

3. Clinton Health Access Initiative, Evidence to Policy Initiative, African Leaders Malaria Alliance. Maintaining the gains: the health and economic benefits of sustaining control measures: UCSF Global Health Group; 2011. Available from: <http://globalhealthsciences.ucsf.edu/sites/default/files/content/ghg/e2pi-maintaining-the-gains.pdf>.

4. Dua V, Sharma S, Srivastava A, Sharma V. Bioenvironmental control of industrial malaria at Bharat Heavy Electricals Ltd., Hardwar, India--results of a nine-year study (1987-95). J Am Mosq Control Assoc. 1997;13(3):278-85.

5. Dy FJ. Present status of malaria control in Asia. Bull World Health Organ. 1954;11(4-5):725-63.

6. Ebi KL. Adaptation costs for climate change-related cases of diarrhoeal disease, malnutrition, and malaria in 2030. Global Health. 2008;4:9.

7. Giron SL, Mateus JC, Castellar CE. Análisis de costo-efectividad de dos intervenciones pala el control de la malaria en el área urbana de Buenaventura, Colombia. Biomedica : revista del Instituto Nacional de Salud. 2006;26(3):379-86.

8. Gunaratna LF. Recent antimalaria work in Ceylon. Bull World Health Organ. 1956;15(3-5):791-9.

9. Haque U, Overgaard HJ, Clements AC, Norris DE, Islam N, Karim J, et al. Malaria burden and control in Bangladesh and prospects for elimination: an epidemiological and economic assessment. Lancet Glob Health. 2014;2(2):e98-e105.

10. Hedman P, Brohult J, Forslund J, Sirleaf V, Bengtsson E. A pocket of controlled malaria in a holoendemic region of West Africa. Ann Trop Med Parasitol. 1979;73(4):317-25.

11. James SP. First Report of the anti-malarial operations at Mian Mir, 1901-1903: Office of the Superintendent of Government Printing, India; 1903.

12. Jowett M, Miller NJ. The financial burden of malaria in Tanzania: implications for future government policy. Int J Health Plann Manage. 2005;20(1):67-84.

13. Kaewsonthi S, Harding AG. The economics of malaria control in Thailand. Parasitol Today. 1989;5(12):392-6.

14. Kamolratanakul P, Butraporn P, Prasittisuk C, Prasittisuk M, Indaratna K. Cost and performance of malaria sector: a case study at Malaria Sector 11, Tak Province, Thailand. Southeast Asian J Trop Med Public Health. 1999;30(3):421-6.

15. Kiszewski A, Johns B, Schapira A, Delacollette C, Crowell V, Tan-Torres T, et al. Estimated global resources needed to attain international malaria control goals. Bull World Health Organ. 2007;85(8):623-30.

16. Kligler IJ. Malaria control demonstrations in Palestine. I. malaria control and its cost. Am J Trop Med Hyg. 1924;1(2):139-74.

17. Kondrashin AV. Malaria in the WHO Southeast Asia region. Indian J Malariol. 1992;29(3):129-60.

18. Konradsen F, Steele P, Perera D, van der Hoek W, Amerasinghe PH, Amerasinghe FP. Cost of malaria control in Sri Lanka. Bull World Health Organ. 1999;77(4):301-9.

19. Korenromp EL, Hosseini M, Newman RD, Cibulskis RE. Progress towards malaria control targets in relation to national malaria programme funding. Malar J. 2013;12:18.

20. Mills A. The economic evaluation of malaria control technologies: the case of Nepal. Soc Sci Med. 1992;34(9):965-72.

21. Mills A. Is malaria control a priority? Evidence from Nepal. Health Econ. 1993;2(4):333-47.

22. Morel CM, Lauer JA, Evans DB. Cost effectiveness analysis of strategies to combat malaria in developing countries. BMJ. 2005;331(7528):1299.

23. Prakash A, Bhattacharyya DR, Mohapatra PK, Barua U, Phukan A, Mahanta J. Malaria control in a forest camp in an oil exploration area of Upper Assam. Natl Med J India. 2003;16(3):135-8.

24. Ramaiah T. Cost benefit analysis of malaria control and eradication programme in India. Ahmedabad: Public Systems Group, Indian Institute of Management; 1980.

25. Ruberu P. Economic justification of intensive malaria control programme in Sri Lanka 1977/81. 1977.

26. Sharma VP. Malaria: cost to India and future trends. Southeast Asian J Trop Med Public Health. 1996;27(1):4-14.

27. Snow RW, Guerra CA, Mutheu JJ, Hay SI. International funding for malaria control in relation to populations at risk of stable Plasmodium falciparum transmission. PLoS Med. 2008;5(7):e142.

28. Some ES. Effects and control of highland malaria epidemic in Uasin Gishu District, Kenya. East Afr Med J. 1994;71(1):2-8.

29. Stuckey EM, Stevenson J, Galactionova K, Baidjoe AY, Bousema T, Odongo W, et al. Modeling the cost effectiveness of malaria control interventions in the highlands of Western kenya. PLoS One. 2014;9(10):e107700.

30. Teklehaimanot A, McCord GC, Sachs JD. Scaling up malaria control in Africa: an economic and epidemiological assessment. Am J Trop Med Hyg. 2007;77(6 Suppl):138-44.

31. Utzinger J, Tozan Y, Doumani F, Singer BH. The economic payoffs of integrated malaria control in the Zambian copperbelt between 1930 and 1950. Trop Med Int Health. 2002;7(8):657-77.

32. Yadav RS, Ghosh SK, Chand SK, Kumar A. Prevalence of malaria and economic loss in two major iron ore mines in Sundargarh district, Orissa. Indian J Malariol. 1991;28(2):105-13.

33. Beaver C. Application of a remoteness index: funding malaria programs. International Journal of Geoinformatics. 2011;7(1).

34. Cohn EJ. Assessing the costs and benefits of anti-malaria programs: the Indian experience. Am J Public Health. 1973;63(12):1086-96.

35. de Zulueta J, Muir DA. Malaria eradication in the Near East. Trans R Soc Trop Med Hyg. 1972;66(5):679-96.

36. Jackson S, Sleigh AC, Liu XL. Cost of malaria control in China: Henan's consolidation programme from community and government perspectives. Bull World Health Organ. 2002;80(8):653-9.

37. Kahn JG, Basu S, Boyle C, Hsiang MS, Jamison DT, Smith Gueye C, et al. Financing elimination. In: Feachem RGA, Phillips AA, Targett GA, editors. Shrinking the malaria map: a prospectus on malaria elimination. San Francisco: The Global Health Group, UCSF Global Health Sciences; 2009.

38. Kahn JG, Hsiang MS, Jamison D. Cost anlysis of malaria elimination in Hainan and Jiangsu Provinces, China and in Swaziland. San Francisco, CA: 2009.

39. Kaneko A, Taleo G, Kalkoa M, Yamar S, Kobayakawa T, Bjorkman A. Malaria eradication on islands. Lancet. 2000;356(9241):1560-4.

40. Liu JX, Newby G, Brackery A, Smith Gueye C, Candari CJ, Escubil LR, et al. Determinants of malaria program expenditures during elimination: case study evidence from select provinces in the Philippines. PLoS One. 2013;8(9):e73352.

41. Livadas G, Athanassatos D. The economic benefits of malaria rradication in Greece. Riv Malariol. 1963;42:177-87.

42. Lok CK. The antimalaria programme in Singapore, with special reference to the period 1974 - 1978. Asian J Infect Dis. 1979;3(1):1-18.

43. Mills A, Lubell Y, Hanson K. Malaria eradication: the economic, financial and institutional challenge. Malar J. 2008;7 Suppl 1:S11.

44. Moonasar D, Morris N, Kleinschmidt I, Maharaj R, Raman J, Mayet NT, et al. What will move malaria control to elimination in South Africa? SAMJ: South African Medical Journal. 2013;103:801-6.

45. Niazi AD. Approximate estimates of the economic loss caused by malaria with some estimates of the benefits of M.E.P. in Iraq. Bull Endem Dis (Baghdad). 1969;11(1):28-39.

46. Ortiz JR. Estimación del costo de un programa de erradicación del paludismo. Bol Oficina Sanit Panam. 1968;64(2):110-5.

47. Purdy M, Robinson M, Wei K, Rublin D. The economic case for combating malaria. Am J Trop Med Hyg. 2013;89(5):819-23.

48. Rezaei-Hemami M, Akbari-Sari A, Raiesi A, Vatandoost H, Majdzadeh R. Cost effectiveness of malaria interventions from preelimination trough elimination: a study in Iran. Journal of Arthropod-Borne Diseases. 2013;8(1):43.

49. Sabot O, Cohen JM, Hsiang MS, Kahn JG, Basu S, Tang L, et al. Costs and financial feasibility of malaria elimination. Lancet. 2010;376(9752):1604-15.

50. Suarez Torres G. El programa de erradicación del paludismo: plan de seis años. Salud Publica Mex. 1970;12(6):751-73.

51. Suarez Torres G. El programa de erradicación del paludismo: resumen del plan con incremento regional de operaciones en parte de la vertiente del Golfo de México y en la Península de Yucatán. Salud Publica Mex. 1970;12(6):745-50.

52. Taiwan Provincial Malaria Research Institute, WHO Malaria Team in Taiwan. Malaria control and eradication in Taiwan: progress report, May 1952 50 June 1957. Bull World Health Organ. 1958;19(4):595-620.

53. Tatarsky A, Aboobakar S, Cohen JM, Gopee N, Bheecarry A, Moonasar D, et al. Preventing the reintroduction of malaria in Mauritius: a programmatic and financial assessment. PLoS One. 2011;6(9):e23832.

1. Asterisks in this column describe whether a study explicitly considered malaria severity, where * = uncomplicated and ** = uncomplicated and severe. [↑](#footnote-ref-1)
2. Unless otherwise stated, the total costs are based on the study period. [↑](#footnote-ref-2)
3. Unless otherwise stated, the costs reported here are the annual costs per capita (i.e., annual total costs of program divided by total population in area of implementation). [↑](#footnote-ref-3)
4. Unless otherwise stated, the costs reported here are the annual costs per PAR (i.e., annual total costs of program divided by PAR in area of implementation). For many studies, the cost per PAR is the same as the cost per capita because the entire population is deemed at risk for malaria. [↑](#footnote-ref-4)
5. Calculated by authors based on total population or PAR reported in the original study. [↑](#footnote-ref-5)
6. Estimated under different scenarios [↑](#footnote-ref-6)
7. No reliable exchange rate could be found for Indian rupees for the years 1901-1902 [↑](#footnote-ref-7)
8. Cost analyses limited to 49 countries outside Africa [↑](#footnote-ref-8)
9. Higher costs are from lower receptive areas (API of 10 and 40 per 1000) while lower costs are from moderate receptive areas (API of 50 and 250 per 1000). [↑](#footnote-ref-9)
10. Values deflated by remoteness and incapacity indices [↑](#footnote-ref-10)
11. Updated costs from (1) Griffith ME. Financial implications of surveillance in India and other countries. *Bulletin of the National Society of India for Malaria and Other Mosquito-borne Diseases* 1961;9:385-411 and (2) Kaewsonthi S, Harding AG. Cost and performance of malaria surveillance in Thailand. *Soc Sci Med* 1992;34(9):1081-1097. [↑](#footnote-ref-11)
12. 2013 costs are based on the exchange rate for New Taiwan dollars (TWD) in the 1950s, which was 5 TWD to 1 USD (see Li K-T. *The evolution of policy behind Taiwan’s development success*. Singapore: World Scientific Publishing Co. Pte. Ltd.) [↑](#footnote-ref-12)
