# Supplementary material for: The economics of malaria control and elimination: a systematic review
Source: Malar J. 2016 Dec 12;15:593. doi: 10.1186/s12936-016-1635-5 (PMC5154116; doi:10.1186/s12936-016-1635-5)
Supplement: Supplementary file 2 — Additional file 2: Table S2. Cost-benefit analyses of malaria control and elimination. [file 12936_2016_1635_MOESM2_ESM.docx]

**S2 Table. Cost-benefit analyses of malaria control and elimination**

| **Source**^[[1]](#footnote-1)^ | **Country or region** | **Study period** | **Perspective/**  **Data sources** | **Costs included** | **Benefits included** | **Total cost (2013 US$)** | **Total benefits (2013 US$)** | **Net benefits (NB, in 2013 US$) or benefit to cost ratio (BCR)** |
| --- | --- | --- | --- | --- | --- | --- | --- | --- |
| Barlow et al. (1986)**^[[2]](#footnote-2)^ [1] | Multiple countries | Varies by country | Societal/  Published literature | Direct cost of control interventions | Economic benefit of increased labor productivity | None | None | *BCR*  Pakistan: 4.9  Philippines: 2.4  Sri Lanka: 146.3  Sudan: 4.6  Thailand: 6.5 |
| Clinton Health Access Initiative, et al. (2011) [2] | Zambia (Konkola copper industry) | 2006-2015 | Societal/  Published data | Direct cost of control interventions | Cost savings | 4,180,249 | 167,733,891 | NB: 163,653,642 BCR: 40 |
| Prakash et al. (2003)** [3] | India (Jorajan camp of Oil India, upper Assam) | April 2000-May 2001 | Societal/  Oil India data | Direct cost of control interventions | Cases averted | 2,746 | 11,387 | NB: 8,644  BCR: 4.14^[[3]](#footnote-3)^ |
| Ramaiah (1980) [4] | India | 1953-1954, 1976-1977 | Societal/  Literature, public sector expenditure reports | Direct and indirect costs of control interventions | Cost savings | 4.274 M | NPV: 39.628 M | BCR: 9.27 |
| Utzinger et al. (2002) [5] | Zambia (4 communities) | 1929-1949 | Societal/  Census data, life tables, literature search, program budgets for control | Direct and indirect costs of control interventions | DALYs averted | 17.1 M | 9.9 M | BCR: 0.573 |
| Livadas et al. (1963) [6] | Greece | 1946-1949 | Societal/  Unspecified | Direct cost of elimination interventions | Cost savings | 11 M | 199 M | NB: 188 M  BCR: 17.093 |
| Mills (2008)2 [7] | Sub-Saharan Africa^[[4]](#footnote-4)^ | Varies | Societal/  Published literature | Direct cost of intensified control interventions | Macro-economic benefits and monetized value of averted DALYs | None | None | *BCR*  Based on macroeconomic benefit: 1.9-4.7  Based on averted DALYs: 17.1 |
| Niazi (1969) [8] | Iraq | 1958-1967 | Societal/  Unspecified | Direct cost of elimination interventions | Cost savings | 86,653,366 | 548,383,410 | NB: 461,425,993  BCR: 6.33 |
| Ortiz (1968) [9] | Paraguay (agricultural and forestry industries) | 1965 | Societal/  Data from Servicio Nacional de Erradicación del Paludismo | Direct and indirect costs of elimination interventions | Increased productivity | 48 M | 139.7 M-220.8 M | BCR: 2.6-3.33 |
| Purdy et al. (2013) [10] | WHO regions | 2010-2030 | Societal/  GMAP | GMAP costs including prevention, case management, program, and R&D | DALYs averted, work years saved, and projected productivity growth | 7.534 M (2010)  7.163 M (2015)  6.338 M (2020)  6.036 M (2025)  4.167 M (2030)  2.877 M (2035) | Several reported based on GDP per person and projected productivity growth | NB (2013-2035): 208.6 B  BCR (2035): 6.113 |

**Note:** The color scheme in the table represents the focus of each study, where intensive malaria control is white and malaria elimination and eradication are in grey.

**Acronyms used in this table**:

B – Billion

BCR – Benefit-cost ratio

DALY – Disability-adjusted life year

M – Million

NB – Net benefit

NPV – Net present value

GDP – Gross domestic product

GMAP – Global Malaria Action Plan

R&D – Research and development

WHO – World Health Organization

**References**:

1. Barlow R, Grobar LM. Costs and benefits of controlling parasitic diseases. Costs and benefits of controlling parasitic diseases: University of Michigan; 1986.

2. Clinton Health Access Initiative, Evidence to Policy Initiative, African Leaders Malaria Alliance. Maintaining the gains: the health and economic benefits of sustaining control measures: UCSF Global Health Group; 2011. Available from: <http://globalhealthsciences.ucsf.edu/sites/default/files/content/ghg/e2pi-maintaining-the-gains.pdf>.

3. Prakash A, Bhattacharyya DR, Mohapatra PK, Barua U, Phukan A, Mahanta J. Malaria control in a forest camp in an oil exploration area of Upper Assam. Natl Med J India. 2003;16(3):135-8.

4. Ramaiah T. Cost benefit analysis of malaria control and eradication programme in India. Ahmedabad: Public Systems Group, Indian Institute of Management; 1980.

5. Utzinger J, Tozan Y, Doumani F, Singer BH. The economic payoffs of integrated malaria control in the Zambian copperbelt between 1930 and 1950. Trop Med Int Health. 2002;7(8):657-77.

6. Livadas G, Athanassatos D. The economic benefits of malaria rradication in Greece. Riv Malariol. 1963;42:177-87.

7. Mills A, Lubell Y, Hanson K. Malaria eradication: the economic, financial and institutional challenge. Malar J. 2008;7 Suppl 1:S11.

8. Niazi AD. Approximate estimates of the economic loss caused by malaria with some estimates of the benefits of M.E.P. in Iraq. Bull Endem Dis (Baghdad). 1969;11(1):28-39.

9. Ortiz JR. Estimación del costo de un programa de erradicación del paludismo. Bol Oficina Sanit Panam. 1968;64(2):110-5.

10. Purdy M, Robinson M, Wei K, Rublin D. The economic case for combating malaria. Am J Trop Med Hyg. 2013;89(5):819-23.

1. Asterisks in this column describe whether a study considered malaria severity, where * = uncomplicated and ** = uncomplicated and severe. [↑](#footnote-ref-1)
2. Review article – only selected studies or findings were extracted and included here [↑](#footnote-ref-2)
3. Calculated by authors based on reported benefits and costs [↑](#footnote-ref-3)
4. More studies conducted in other studies are included in the original review article, but only the BCRs from Sub-Saharan Africa are reported here. Findings from other studies in Sudan, Thailand, Pakistan, Greece, Sri Lanka, Iraq, Paraguay, and India are reported separately in this table or are included in the Barlow et al. (1986) entry. [↑](#footnote-ref-4)
