# Supplementary material for: The economics of malaria control and elimination: a systematic review
Source: Malar J. 2016 Dec 12;15:593. doi: 10.1186/s12936-016-1635-5 (PMC5154116; doi:10.1186/s12936-016-1635-5)
Supplement: Supplementary file 3 — Additional file 3: Table S3. Quality assessment of CBAs. [file 12936_2016_1635_MOESM3_ESM.docx]

**S3 Table: Quality assessment of cost-benefit analyses using the 10-point Drummond checklist**

| Article | Q1 | Q2 | Q3 | Q4 | Q5 | Q6 | Q7 | Q8 | Q9 | Q10 |  |
| --- | --- | --- | --- | --- | --- | --- | --- | --- | --- | --- | --- |
|  | Was a well-defined question posed in answerable form? | Was a comprehensive description of the competing alternatives given? | Was the effectiveness of the programmes or services established? | Were all the important and relevant costs and consequences for each alternative identified? | Were costs and consequences measured accurately in appropriate physical units? | Were costs and consequences valued credibly? | Were costs and consequences adjusted for differential timing? | Was an incremental analysis of costs and consequences of alternatives performed? | Was allowance made for uncertainty in the estimates of costs and consequences? | Did the presentation and discussion of study results include all issues of concern to users? | **TOTAL SCORE** |
| Barlow et al. (1986)^*^ [1] | NA | NA | NA | NA | NA | NA | NA | NA | NA | NA | NA |
| Clinton Health Access Initiative, et al. (2011) [2] | Y | Y | Y | N | Y | Y | N | Y | N | N | 6 |
| Prakash et al. (2003) [3] | Y | Y | Y | N | N | N | N | N | N | N | 3 |
| Ramaiah (1980) [4] | Y | Y | Y | N | Y | Y | N | Y | N | N | 6 |
| Utzinger et al. (2002) [5] | Y | Y | Y | Y | Y | Y | Y | N | Y | Y | 9 |
| Livadas et al. (1963) [6] | N | Y | N | N | N | N | N | N | N | N | 1 |
| Mills (2008)^*^ [7] | NA | NA | NA | NA | NA | NA | NA | NA | NA | NA | NA |
| Niazi (1969) [8] | Y | Y | N | N | N | N | N | Y | N | N | 3 |
| Ortiz (1968) [9] | N | Y | Y | N | N | Y | N | N | N | N | 3 |
| Purdy et al. (2013) [10] | Y | Y | Y | N | Y | Y | Y | Y | N | N | 7 |

**Note**: The color scheme in the table represents the focus of each study, where intensive malaria control is white and malaria elimination and eradication are in grey.

* These articles are reviews, which could not be assessed for quality using the Drummond checklist.

**References**:

1. Barlow R, Grobar LM. Costs and benefits of controlling parasitic diseases. Costs and benefits of controlling parasitic diseases: University of Michigan; 1986.

2. Clinton Health Access Initiative, Evidence to Policy Initiative, African Leaders Malaria Alliance. Maintaining the gains: the health and economic benefits of sustaining control measures: UCSF Global Health Group; 2011. Available from: <http://globalhealthsciences.ucsf.edu/sites/default/files/content/ghg/e2pi-maintaining-the-gains.pdf>.

3. Prakash A, Bhattacharyya DR, Mohapatra PK, Barua U, Phukan A, Mahanta J. Malaria control in a forest camp in an oil exploration area of Upper Assam. Natl Med J India. 2003;16(3):135-8.

4. Ramaiah T. Cost benefit analysis of malaria control and eradication programme in India. Ahmedabad: Public Systems Group, Indian Institute of Management; 1980.

5. Utzinger J, Tozan Y, Doumani F, Singer BH. The economic payoffs of integrated malaria control in the Zambian copperbelt between 1930 and 1950. Trop Med Int Health. 2002;7(8):657-77.

6. Livadas G, Athanassatos D. The economic benefits of malaria rradication in Greece. Riv Malariol. 1963;42:177-87.

7. Mills A, Lubell Y, Hanson K. Malaria eradication: the economic, financial and institutional challenge. Malar J. 2008;7 Suppl 1:S11.

8. Niazi AD. Approximate estimates of the economic loss caused by malaria with some estimates of the benefits of M.E.P. in Iraq. Bull Endem Dis (Baghdad). 1969;11(1):28-39.

9. Ortiz JR. Estimación del costo de un programa de erradicación del paludismo. Bol Oficina Sanit Panam. 1968;64(2):110-5.

10. Purdy M, Robinson M, Wei K, Rublin D. The economic case for combating malaria. Am J Trop Med Hyg. 2013;89(5):819-23.
