# Supplementary material for: The economics of malaria control and elimination: a systematic review
Source: Malar J. 2016 Dec 12;15:593. doi: 10.1186/s12936-016-1635-5 (PMC5154116; doi:10.1186/s12936-016-1635-5)
Supplement: Supplementary file 4 — Additional file 4: Table S4. Quality assessment of costing analyses. [file 12936_2016_1635_MOESM4_ESM.docx]

**S4 Table: Quality assessment of costing studies**

| **Reference** | **Scope of costing** | | **Accuracy of method evaluating cost** | |
| --- | --- | --- | --- | --- |
| Abeyasinghe et al. (2012) [1] | B | All components of costs were described and data for costs in each component were reported. | α | Micro-costing estimates based on individual item expenses and/or detailed data sets |
| Akhavan et al. (1999) [2] | B | All components of costs were described and data for costs in each component were reported. | γ | Use of charge data as a proxy |
| Beaver (2011) [3] | D | Only scope of costing was described but components of costs were not described. | δ | No clear description of cost accounting methods or inability to confirm the method easily (i.e., data are taken from budget or financial reports, published or unpublished literature, personal communication, and other secondary sources) |
| Clinton Health Access Initiative, et al. (2011) [4] | A | All components of costs were described and data for both quantity and unit price of resources were reported for each component. | γ | Use of charge data as a proxy |
| Cohn (1973) [5] | D | Only scope of costing was described but components of costs were not described. | δ | No clear description of cost accounting methods or inability to confirm the method easily (i.e., data are taken from budget or financial reports, published or unpublished literature, personal communication, and other secondary sources) |
| de Zulueta et al. (1972) [6] | D | Only scope of costing was described but components of costs were not described. | δ | No clear description of cost accounting methods or inability to confirm the method easily (i.e., data are taken from budget or financial reports, published or unpublished literature, personal communication, and other secondary sources) |
| Dua et al. (1997) [7] | D | Only scope of costing was described but components of costs were not described. | δ | No clear description of cost accounting methods or inability to confirm the method easily (i.e., data are taken from budget or financial reports, published or unpublished literature, personal communication, and other secondary sources) |
| Dy (1954) [8] | B | All components of costs were described and data for costs in each component were reported. | δ | No clear description of cost accounting methods or inability to confirm the method easily (i.e., data are taken from budget or financial reports, published or unpublished literature, personal communication, and other secondary sources) |
| Ebi (2008) [9] | C | All components of costs were described but costs in each component were not reported. | γ | Use of charge data as a proxy |
| Giron et al. (2006) [10] | C | All components of costs were described but costs in each component were not reported. | δ | No clear description of cost accounting methods or inability to confirm the method easily (i.e., data are taken from budget or financial reports, published or unpublished literature, personal communication, and other secondary sources) |
| Gunaratna (1956) [11] | D | Only scope of costing was described but components of costs were not described. | δ | No clear description of cost accounting methods or inability to confirm the method easily (i.e., data are taken from budget or financial reports, published or unpublished literature, personal communication, and other secondary sources) |
| Haque et al. (2014) [12] | D | Only scope of costing was described but components of costs were not described. | δ | No clear description of cost accounting methods or inability to confirm the method easily (i.e., data are taken from budget or financial reports, published or unpublished literature, personal communication, and other secondary sources) |
| Hedman et al. (1979) [13] | A | All components of costs were described and data for both quantity and unit price of resources were reported for each component. | β | Estimates based on relative value units (RVUs) or ratio of costs to charges (RCCs) |
| Jackson et al. (2002) [14] | B | All components of costs were described and data for costs in each component were reported. | α | Micro-costing estimates based on individual item expenses and/or detailed data sets |
| James (1903) [15] | B | All components of costs were described and data for costs in each component were reported. | δ | No clear description of cost accounting methods or inability to confirm the method easily (i.e., data are taken from budget or financial reports, published or unpublished literature, personal communication, and other secondary sources) |
| Jowett et al. (2005) [16] | B | All components of costs were described and data for costs in each component were reported. | γ | Use of charge data as a proxy |
| Kaewsonthi et al. (1989) [17] | B | All components of costs were described and data for costs in each component were reported. | δ | No clear description of cost accounting methods or inability to confirm the method easily (i.e., data are taken from budget or financial reports, published or unpublished literature, personal communication, and other secondary sources) |
| Kahn et al. (2009a) [18] | D | Only scope of costing was described but components of costs were not described. | δ | No clear description of cost accounting methods or inability to confirm the method easily (i.e., data are taken from budget or financial reports, published or unpublished literature, personal communication, and other secondary sources) |
| Kahn et al. (2009b) [19] | D | Only scope of costing was described but components of costs were not described. | δ | No clear description of cost accounting methods or inability to confirm the method easily (i.e., data are taken from budget or financial reports, published or unpublished literature, personal communication, and other secondary sources) |
| Kamolratanakul et al. (1999) [20] | B | All components of costs were described and data for costs in each component were reported. | δ | No clear description of cost accounting methods or inability to confirm the method easily (i.e., data are taken from budget or financial reports, published or unpublished literature, personal communication, and other secondary sources) |
| Kaneko et al. (2000) [21] | B | All components of costs were described and data for costs in each component were reported. | δ | No clear description of cost accounting methods or inability to confirm the method easily (i.e., data are taken from budget or financial reports, published or unpublished literature, personal communication, and other secondary sources) |
| Kiszewski et al. (2007) [22] | A | All components of costs were described and data for both quantity and unit price of resources were reported for each component. | γ | Use of charge data as a proxy |
| Kligler (1924) [23] | B | All components of costs were described and data for costs in each component were reported. | δ | No clear description of cost accounting methods or inability to confirm the method easily (i.e., data are taken from budget or financial reports, published or unpublished literature, personal communication, and other secondary sources) |
| Kondrashin (1992) [24] | D | Only scope of costing was described but components of costs were not described. | δ | No clear description of cost accounting methods or inability to confirm the method easily (i.e., data are taken from budget or financial reports, published or unpublished literature, personal communication, and other secondary sources) |
| Konradsen et al. (1999) [25] | B | All components of costs were described and data for costs in each component were reported. | α | Micro-costing estimates based on individual item expenses and/or detailed data sets |
| Korenromp et al. (2013) [26] | D | Only scope of costing was described but components of costs were not described. | δ | No clear description of cost accounting methods or inability to confirm the method easily (i.e., data are taken from budget or financial reports, published or unpublished literature, personal communication, and other secondary sources) |
| Liu et al. (2013) [27] | A | All components of costs were described and data for both quantity and unit price of resources were reported for each component. | α | Micro-costing estimates based on individual item expenses and/or detailed data sets |
| Livadas et al. (1963) [28] | D | Only scope of costing was described but components of costs were not described. | δ | No clear description of cost accounting methods or inability to confirm the method easily (i.e., data are taken from budget or financial reports, published or unpublished literature, personal communication, and other secondary sources) |
| Lok (1979) [29] | B | All components of costs were described and data for costs in each component were reported. | δ | No clear description of cost accounting methods or inability to confirm the method easily (i.e., data are taken from budget or financial reports, published or unpublished literature, personal communication, and other secondary sources) |
| Mills (1992) [30] | D | Only scope of costing was described but components of costs were not described. | δ | No clear description of cost accounting methods or inability to confirm the method easily (i.e., data are taken from budget or financial reports, published or unpublished literature, personal communication, and other secondary sources) |
| Mills (1993b) [31] | B | All components of costs were described and data for costs in each component were reported. | β | Estimates based on relative value units (RVUs) or ratio of costs to charges (RCCs) |
| Mills (2008)* [32] | NA | Assessment not applicable | NA | Assessment not applicable |
| Moonasar et al. (2013) [33] | B | All components of costs were described and data for costs in each component were reported. | δ | No clear description of cost accounting methods or inability to confirm the method easily (i.e., data are taken from budget or financial reports, published or unpublished literature, personal communication, and other secondary sources) |
| Morel et al. (2005) [34] | B | All components of costs were described and data for costs in each component were reported. | β | Estimates based on relative value units (RVUs) or ratio of costs to charges (RCCs) |
| Niazi (1969) [35] | A | All components of costs were described and data for both quantity and unit price of resources were reported for each component. | δ | No clear description of cost accounting methods or inability to confirm the method easily (i.e., data are taken from budget or financial reports, published or unpublished literature, personal communication, and other secondary sources) |
| Ortiz (1968) [36] | D | Only scope of costing was described but components of costs were not described. | δ | No clear description of cost accounting methods or inability to confirm the method easily (i.e., data are taken from budget or financial reports, published or unpublished literature, personal communication, and other secondary sources) |
| Prakash et al. (2003) [37] | B | All components of costs were described and data for costs in each component were reported. | δ | No clear description of cost accounting methods or inability to confirm the method easily (i.e., data are taken from budget or financial reports, published or unpublished literature, personal communication, and other secondary sources) |
| Purdy et al. (2013) [38] | D | Only scope of costing was described but components of costs were not described. | δ | No clear description of cost accounting methods or inability to confirm the method easily (i.e., data are taken from budget or financial reports, published or unpublished literature, personal communication, and other secondary sources) |
| Ramaiah (1980) [39] | D | Only scope of costing was described but components of costs were not described. | δ | No clear description of cost accounting methods or inability to confirm the method easily (i.e., data are taken from budget or financial reports, published or unpublished literature, personal communication, and other secondary sources) |
| Rezaei-Hemami et al. (2014) [40] | B | All components of costs were described and data for costs in each component were reported. | δ | No clear description of cost accounting methods or inability to confirm the method easily (i.e., data are taken from budget or financial reports, published or unpublished literature, personal communication, and other secondary sources) |
| Ruberu (1977) [41] | B | All components of costs were described and data for costs in each component were reported. | γ | Use of charge data as a proxy |
| Sabot et al. (2010) [42] | A | All components of costs were described and data for both quantity and unit price of resources were reported for each component. | α | Micro-costing estimates based on individual item expenses and/or detailed data sets |
| Sharma (1996) [43] | B | All components of costs were described and data for costs in each component were reported. | δ | No clear description of cost accounting methods or inability to confirm the method easily (i.e., data are taken from budget or financial reports, published or unpublished literature, personal communication, and other secondary sources) |
| Snow et al. (2008) [44] | D | Only scope of costing was described but components of costs were not described. | δ | No clear description of cost accounting methods or inability to confirm the method easily (i.e., data are taken from budget or financial reports, published or unpublished literature, personal communication, and other secondary sources) |
| Some (1994) [45] | B | All components of costs were described and data for costs in each component were reported. | δ | No clear description of cost accounting methods or inability to confirm the method easily (i.e., data are taken from budget or financial reports, published or unpublished literature, personal communication, and other secondary sources) |
| Stuckey et al. (2014) [46] | A | All components of costs were described and data for both quantity and unit price of resources were reported for each component. | γ | Use of charge data as a proxy |
| Suarez Torres (1970a) [47] | B | All components of costs were described and data for costs in each component were reported. | δ | No clear description of cost accounting methods or inability to confirm the method easily (i.e., data are taken from budget or financial reports, published or unpublished literature, personal communication, and other secondary sources) |
| Suarez Torres (1970b) [48] | D | Only scope of costing was described but components of costs were not described. | δ | No clear description of cost accounting methods or inability to confirm the method easily (i.e., data are taken from budget or financial reports, published or unpublished literature, personal communication, and other secondary sources) |
| Taiwan Provincial Malaria Research Institute et al. (1958) [49] | D | Only scope of costing was described but components of costs were not described. | δ | No clear description of cost accounting methods or inability to confirm the method easily (i.e., data are taken from budget or financial reports, published or unpublished literature, personal communication, and other secondary sources) |
| Tatarsky et al. (2011) [50] | A | All components of costs were described and data for both quantity and unit price of resources were reported for each component. | α | Micro-costing estimates based on individual item expenses and/or detailed data sets |
| Teklehaimanot et al. (2007) [51] | B | All components of costs were described and data for costs in each component were reported. | δ | No clear description of cost accounting methods or inability to confirm the method easily (i.e., data are taken from budget or financial reports, published or unpublished literature, personal communication, and other secondary sources) |
| Utzinger et al. (2002) [52] | C | All components of costs were described but costs in each component were not reported. | δ | No clear description of cost accounting methods or inability to confirm the method easily (i.e., data are taken from budget or financial reports, published or unpublished literature, personal communication, and other secondary sources) |
| Yadav et al. (1991) [53] | B | All components of costs were described and data for costs in each component were reported. | γ | Use of charge data as a proxy |

**Note**: References in RED are in Spanish or French.

* These articles are reviews, which could not be assessed for quality using the Fukuda and Imanaka checklist.

**References**:

1. Abeyasinghe RR, Galappaththy GN, Smith Gueye C, Kahn JG, Feachem RG. Malaria control and elimination in Sri Lanka: documenting progress and success factors in a conflict setting. PLoS One. 2012;7(8):e43162.

2. Akhavan D, Musgrove P, Abrantes A, d AGR. Cost-effective malaria control in Brazil: cost-effectiveness of a malaria control program in the Amazon Basin of Brazil, 1988-1996. Soc Sci Med. 1999;49(10):1385-99.

3. Beaver C. Application of a remoteness index: funding malaria programs. International Journal of Geoinformatics. 2011;7(1).

4. Clinton Health Access Initiative, Evidence to Policy Initiative, African Leaders Malaria Alliance. Maintaining the gains: the health and economic benefits of sustaining control measures: UCSF Global Health Group; 2011. Available from: <http://globalhealthsciences.ucsf.edu/sites/default/files/content/ghg/e2pi-maintaining-the-gains.pdf>.

5. Cohn EJ. Assessing the costs and benefits of anti-malaria programs: the Indian experience. Am J Public Health. 1973;63(12):1086-96.

6. de Zulueta J, Muir DA. Malaria eradication in the Near East. Trans R Soc Trop Med Hyg. 1972;66(5):679-96.

7. Dua V, Sharma S, Srivastava A, Sharma V. Bioenvironmental control of industrial malaria at Bharat Heavy Electricals Ltd., Hardwar, India--results of a nine-year study (1987-95). J Am Mosq Control Assoc. 1997;13(3):278-85.

8. Dy FJ. Present status of malaria control in Asia. Bull World Health Organ. 1954;11(4-5):725-63.

9. Ebi KL. Adaptation costs for climate change-related cases of diarrhoeal disease, malnutrition, and malaria in 2030. Global Health. 2008;4:9.

10. Giron SL, Mateus JC, Castellar CE. Análisis de costo-efectividad de dos intervenciones pala el control de la malaria en el área urbana de Buenaventura, Colombia. Biomedica : revista del Instituto Nacional de Salud. 2006;26(3):379-86.

11. Gunaratna LF. Recent antimalaria work in Ceylon. Bull World Health Organ. 1956;15(3-5):791-9.

12. Haque U, Overgaard HJ, Clements AC, Norris DE, Islam N, Karim J, et al. Malaria burden and control in Bangladesh and prospects for elimination: an epidemiological and economic assessment. Lancet Glob Health. 2014;2(2):e98-e105.

13. Hedman P, Brohult J, Forslund J, Sirleaf V, Bengtsson E. A pocket of controlled malaria in a holoendemic region of West Africa. Ann Trop Med Parasitol. 1979;73(4):317-25.

14. Jackson S, Sleigh AC, Liu XL. Cost of malaria control in China: Henan's consolidation programme from community and government perspectives. Bull World Health Organ. 2002;80(8):653-9.

15. James SP. First Report of the anti-malarial operations at Mian Mir, 1901-1903: Office of the Superintendent of Government Printing, India; 1903.

16. Jowett M, Miller NJ. The financial burden of malaria in Tanzania: implications for future government policy. Int J Health Plann Manage. 2005;20(1):67-84.

17. Kaewsonthi S, Harding AG. The economics of malaria control in Thailand. Parasitol Today. 1989;5(12):392-6.

18. Kahn JG, Basu S, Boyle C, Hsiang MS, Jamison DT, Smith Gueye C, et al. Financing elimination. In: Feachem RGA, Phillips AA, Targett GA, editors. Shrinking the Malaria Map. San Francisco, CA: The Global Health Group; 2009. p. 61-80.

19. Kahn JG, Hsiang MS, Jamison D. Cost anlysis of malaria elimination in Hainan and Jiangsu Provinces, China and in Swaziland. San Francisco, CA: 2009.

20. Kamolratanakul P, Butraporn P, Prasittisuk C, Prasittisuk M, Indaratna K. Cost and performance of malaria sector: a case study at Malaria Sector 11, Tak Province, Thailand. Southeast Asian J Trop Med Public Health. 1999;30(3):421-6.

21. Kaneko A, Taleo G, Kalkoa M, Yamar S, Kobayakawa T, Bjorkman A. Malaria eradication on islands. Lancet. 2000;356(9241):1560-4.

22. Kiszewski A, Johns B, Schapira A, Delacollette C, Crowell V, Tan-Torres T, et al. Estimated global resources needed to attain international malaria control goals. Bull World Health Organ. 2007;85(8):623-30.

23. Kligler IJ. Malaria control demonstrations in Palestine. I. malaria control and its cost. Am J Trop Med Hyg. 1924;1(2):139-74.

24. Kondrashin AV. Malaria in the WHO Southeast Asia region. Indian J Malariol. 1992;29(3):129-60.

25. Konradsen F, Steele P, Perera D, van der Hoek W, Amerasinghe PH, Amerasinghe FP. Cost of malaria control in Sri Lanka. Bull World Health Organ. 1999;77(4):301-9.

26. Korenromp EL, Hosseini M, Newman RD, Cibulskis RE. Progress towards malaria control targets in relation to national malaria programme funding. Malar J. 2013;12:18.

27. Liu JX, Newby G, Brackery A, Smith Gueye C, Candari CJ, Escubil LR, et al. Determinants of malaria program expenditures during elimination: case study evidence from select provinces in the Philippines. PLoS One. 2013;8(9):e73352.

28. Livadas G, Athanassatos D. The economic benefits of malaria rradication in Greece. Riv Malariol. 1963;42:177-87.

29. Lok CK. The antimalaria programme in Singapore, with special reference to the period 1974 - 1978. Asian J Infect Dis. 1979;3(1):1-18.

30. Mills A. The economic evaluation of malaria control technologies: the case of Nepal. Soc Sci Med. 1992;34(9):965-72.

31. Mills A. Is malaria control a priority? Evidence from Nepal. Health Econ. 1993;2(4):333-47.

32. Mills A, Lubell Y, Hanson K. Malaria eradication: the economic, financial and institutional challenge. Malar J. 2008;7 Suppl 1:S11.

33. Moonasar D, Morris N, Kleinschmidt I, Maharaj R, Raman J, Mayet NT, et al. What will move malaria control to elimination in South Africa? SAMJ: South African Medical Journal. 2013;103:801-6.

34. Morel CM, Lauer JA, Evans DB. Cost effectiveness analysis of strategies to combat malaria in developing countries. BMJ. 2005;331(7528):1299.

35. Niazi AD. Approximate estimates of the economic loss caused by malaria with some estimates of the benefits of M.E.P. in Iraq. Bull Endem Dis (Baghdad). 1969;11(1):28-39.

36. Ortiz JR. Estimación del costo de un programa de erradicación del paludismo. Bol Oficina Sanit Panam. 1968;64(2):110-5.

37. Prakash A, Bhattacharyya DR, Mohapatra PK, Barua U, Phukan A, Mahanta J. Malaria control in a forest camp in an oil exploration area of Upper Assam. Natl Med J India. 2003;16(3):135-8.

38. Purdy M, Robinson M, Wei K, Rublin D. The economic case for combating malaria. Am J Trop Med Hyg. 2013;89(5):819-23.

39. Ramaiah T. Cost benefit analysis of malaria control and eradication programme in India. Ahmedabad: Public Systems Group, Indian Institute of Management; 1980.

40. Rezaei-Hemami M, Akbari-Sari A, Raiesi A, Vatandoost H, Majdzadeh R. Cost effectiveness of malaria interventions from preelimination trough elimination: a study in Iran. Journal of Arthropod-Borne Diseases. 2013;8(1):43.

41. Ruberu P. Economic justification of intensive malaria control programme in Sri Lanka 1977/81. 1977.

42. Sabot O, Cohen JM, Hsiang MS, Kahn JG, Basu S, Tang L, et al. Costs and financial feasibility of malaria elimination. Lancet. 2010;376(9752):1604-15.

43. Sharma VP. Malaria: cost to India and future trends. Southeast Asian J Trop Med Public Health. 1996;27(1):4-14.

44. Snow RW, Guerra CA, Mutheu JJ, Hay SI. International funding for malaria control in relation to populations at risk of stable Plasmodium falciparum transmission. PLoS Med. 2008;5(7):e142.

45. Some ES. Effects and control of highland malaria epidemic in Uasin Gishu District, Kenya. East Afr Med J. 1994;71(1):2-8.

46. Stuckey EM, Stevenson J, Galactionova K, Baidjoe AY, Bousema T, Odongo W, et al. Modeling the cost effectiveness of malaria control interventions in the highlands of Western kenya. PLoS One. 2014;9(10):e107700.

47. Suarez Torres G. El programa de erradicación del paludismo: plan de seis años. Salud Publica Mex. 1970;12(6):751-73.

48. Suarez Torres G. El programa de erradicación del paludismo: resumen del plan con incremento regional de operaciones en parte de la vertiente del Golfo de México y en la Península de Yucatán. Salud Publica Mex. 1970;12(6):745-50.

49. Taiwan Provincial Malaria Research Institute, WHO Malaria Team in Taiwan. Malaria control and eradication in Taiwan: progress report, May 1952 50 June 1957. Bull World Health Organ. 1958;19(4):595-620.

50. Tatarsky A, Aboobakar S, Cohen JM, Gopee N, Bheecarry A, Moonasar D, et al. Preventing the reintroduction of malaria in Mauritius: a programmatic and financial assessment. PLoS One. 2011;6(9):e23832.

51. Teklehaimanot A, McCord GC, Sachs JD. Scaling up malaria control in Africa: an economic and epidemiological assessment. Am J Trop Med Hyg. 2007;77(6 Suppl):138-44.

52. Utzinger J, Tozan Y, Doumani F, Singer BH. The economic payoffs of integrated malaria control in the Zambian copperbelt between 1930 and 1950. Trop Med Int Health. 2002;7(8):657-77.

53. Yadav RS, Ghosh SK, Chand SK, Kumar A. Prevalence of malaria and economic loss in two major iron ore mines in Sundargarh district, Orissa. Indian J Malariol. 1991;28(2):105-13.
